# Topical Delivery Systems for Plant-Derived Antimicrobial Agents: A Review of Current Advances

**DOI:** 10.1155/ijbm/4251091

**Published:** 2025-07-27

**Authors:** Mohammad Hashem Hashempur, Fereshteh Ghorat, Forough Karami, Alireza Jahanbin, Hasti Nouraei, Milad Abbasi, Mahboobeh Jafari, Alireza Zare, Sajjad Barzegar, Zahra Zareshahrabadi

**Affiliations:** ^1^Research Center for Traditional Medicine and History of Medicine, Department of Persian Medicine, School of Medicine, Shiraz University of Medical Sciences, Shiraz, Iran; ^2^Non-Communicable Diseases Research Center, Sabzevar University of Medical Sciences, Sabzevar, Iran; ^3^Central Research Laboratory, School of Medicine, Shiraz University of Medical Sciences, Shiraz, Iran; ^4^Department of Materials Science and Engineering, School of Engineering, Shiraz University, Shiraz, Iran; ^5^Department of Parasitology and Mycology, School of Medicine, Shiraz University of Medical Sciences, Shiraz, Iran; ^6^Department of Medical Nanotechnology, School of Advanced Technologies in Medicine, Shiraz University of Medical Sciences, Shiraz, Iran; ^7^Center for Nanotechnology in Drug Delivery, Shiraz University of Medical Sciences, Shiraz, Iran; ^8^Department of Chemical Engineering, Shiraz University, Shiraz, Iran; ^9^Basic Sciences in Infectious Diseases Research Center, Shiraz University of Medical Sciences, Shiraz, Iran

**Keywords:** antimicrobial agents, herbal extract, integrative medicine, nanomaterials, plant-derived compounds, topical delivery, traditional Persian medicine

## Abstract

Plant-derived compounds have attracted considerable attention in the field of antimicrobial therapy. This interest is primarily due to their natural origin and historical evidence of their use in traditional medicine systems. These derivatives are a rich reservoir of chemical diversity that has a promising potential for the development and production of new antimicrobial agents with the least amount of side effects and risks of drug resistance. However, the delivery of plant-derived antimicrobial agents, especially through the topical route, poses significant challenges. As the largest organ of the body, the skin acts as a first barrier against the entrance of microbial pathogens. A primary limitation to transdermal delivery of plant-derived antimicrobial agents is their complex molecular structures, which often prevent effective absorption through the skin. Therefore, developing and promoting an effective local drug delivery system to increase the potential of antimicrobial therapy is very important and effective in public health. This review discusses delivery strategies for plant-derived antimicrobial agents aimed at the bioavailability and stability of these compounds as well as their mode of action, ensuring targeted delivery to the site of infection with long-lasting effects and minimizing side effects. Besides, various topical drug delivery platforms are analyzed, including nanoparticles, liposomes, and innovative application methods such as microneedles.

## 1. Introduction

Antimicrobial drugs play a vital role in treating infectious diseases by preventing and eliminating microbial pathogens [1, 2]. However, their widespread use has led to a major public health concern: antimicrobial resistance (AMR). This occurs when microbe develop mechanisms to evade the effects of antibiotics, rendering treatments ineffective [3]. There have been some mechanisms outlined by which bacterial strains may circumvent the effects of antibiotics: (a) alteration or mutation of the target; (b) decrease in permeability; (c) efflux pumps; (d) hydrolase or inactivating enzyme; (e) metabolic increase or auxotrophy; (f) target protective protein; (g) activation of internal repair mechanisms; (h) cell morphological changes; and (i) cooperative community resistance [4]. If an infective microbe is resistant to many antibiotics, several things can occur. (a) Failed treatment: Lack of therapeutic responsiveness results in increased duration of disease and death hazard. (b) Increased lengths of hospitalization: Longer illness and hospitalizations enable greater secondary spread in the community. (c) Transfer to potent antibiotics: After primary-line failure, therapy is shifted toward more expensive and maybe more risky second- or third-line drugs. (d) Threat to modern medicine: The development of contemporary medicine is being nullified by antibiotic resistance. Without effective antibiotics, operations such as organ transplant, chemotherapy, and surgery become much more risky [5]. Novel nanosized drug delivery systems provide an intriguing solution to overcoming the dominating problems in antibiotic therapy since they possess unique physicochemical properties. These are marked by a large surface area-to-mass ratio, tiny particle size, distinct interactions with host cells and microorganisms, and the ability to be structurally and functionally modified. The benefits of nanosized antibiotic drug delivery systems are targeted delivery, more even distribution in the target tissue, improved cellular uptake and solubility, sustained release of the drug, reduced side effects, and improved patient compliance [6]. Meanwhile, conventional medicine and natural products have also received a great deal of emphasis, which is attributed to past precedent and their contribution to conventional systems of medicine in many cultures around the globe [7, 8]. Natural products present a diverse and voluminous chemical resource of immense potential to develop novel antimicrobial drugs with fewer side effects and averting the threat of drug resistance [7, 9]. The novel drug-like activity coupled with broad-spectrum antimicrobial activity of these plant-derived compounds indicates their potential as effective molecules for microbial strain warfare [10–13]. Even though they demonstrate significant therapeutic potential, delivery of antimicrobial plant agents, particularly transdermal, is highly challenging. Since skin is the largest organ of the body, it acts as a robust barrier in shielding the internal body against foreign invasion by microbial pathogens [14]. Further, its intrinsic barrier properties also hamper effective penetration through it of therapy molecules. This is a very important drawback in the case of plant-derived compounds, most of which have complex molecular structures that are not taken up by the skin [15]. Thus, good topical drug delivery systems need to be developed to obtain the optimal therapeutic effects of these natural antimicrobials. Contemporary drug delivery systems are intended to maximize such drugs' bioavailability and stability, deliver them site specifically to sites of infection, prolong action, and minimize systemic side effects [16, 17]. Employment of antimicrobial plant constituents as part of contemporary drug delivery systems is a novel aspect of topical therapy [9]. Even though phytoconstituents such as phenolics, terpenoids, and essential oils have good antimicrobial activity, their application in a clinical environment is usually plagued by stability, bioavailability, and skin permeability. Recent developments in material science and nanotechnology have allowed the development of sophisticated delivery systems like nanostructured lipid carriers (NLCs) and solid lipid nanoparticles (NPs) (SLNs) (lipid-based NPs), liposomes, hydrogels, and microneedles (MNs) for effective encapsulation and delivery of such bioactive compounds [6, 18, 19]. These delivery systems not only enhance the physicochemical stability of plant antimicrobials but also allow targeted delivery at infection sites with controlled release and mitigation of systemic side effects. By combining traditional plant medicine with modern delivery technologies, this blend offers a novel way of tapping the full therapeutic potential of natural products, particularly in the era of developing antimicrobial resistance [6, 20, 21]. This review aims to evaluate local drug delivery strategies for plant-derived antimicrobial medicines by reviewing the previous literature. Different topical delivery systems have been found to enhance the efficacy of plant-derived antimicrobials like NPs, liposomes, hydrogels, and transdermal patches. In the following sections, limitations and issues of dermal delivery of plant-derived antimicrobials have been discussed.

## 2. Characteristics of Skin and Overview of Drug Transport Mechanisms

Topical and transdermal drug delivery systems (TDDSs) are closely linked to the qualities of the skin and the medication or active pharmaceutical ingredient (API), regardless of the principles and formulations involved.

**Figure 1:**
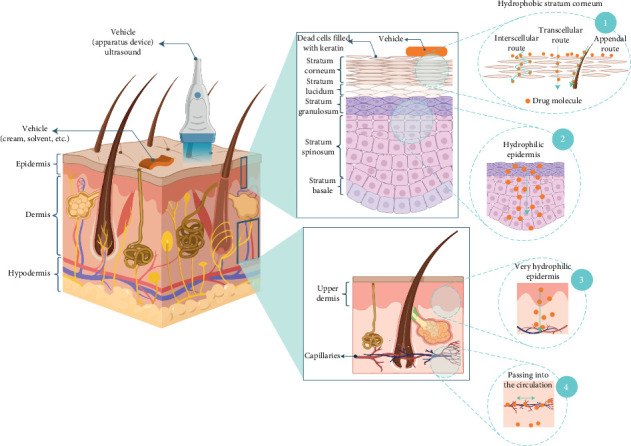
This figure shows how drugs penetrate the skin's layers to achieve therapeutic effects. The skin acts as both a protective barrier and a pathway for drug administration, consisting of three main layers: Epidermis: The outermost layer, made mostly of keratinocytes. Its surface, the stratum corneum, is a tough barrier that restricts most drug entry. Beneath it, the living cells of the viable epidermis depend on the dermis for nutrients. Dermis: The middle layer, rich in connective tissue, blood vessels, nerves, hair follicles, and glands. Drugs reaching this layer can enter the bloodstream for systemic effects. Hypodermis (subcutaneous tissue): the deepest layer, containing fat and larger blood vessels. It insulates the body and connects the skin to muscles and bones. Some drugs target this layer for slow, sustained absorption.

As indicated in Figure 1, the skin is a complicated structure composed of three layers: the epidermis, dermis, and hypodermis. The protective layer serves to exclude external invasions such as microbes and chemicals and also minimizes the loss of internal materials. Permeability of the skin varies with anatomic location, gender, age, moisture, and external temperature, largely as a function of the composition of the stratum corneum, the outermost layer, which is rich in keratin [22]. Transdermal drug delivery involves the penetration of an API via the skin at therapeutic rates. This process, also known as percutaneous absorption, consists of five basic steps. A “carrier,” such as a transdermal apparatus, cream, or solvent, is commonly utilized to include the drug or API. During the first phase, the active molecule is transferred from the carrier to the hydrolipidic film based on the molecule's mobility and partition coefficient [23]. The medicine gets absorbed into a particular layer of the skin, known as the viable epidermis, after penetrating the stratum corneum. From there, the molecule diffuses to the upper dermis. Permeation occurs between layers, allowing the medication to reach the circulation in the absorption or resorption stage [24]. The transfer process is influenced by several elements at each stage and may be defined kinetically in different ways. However, generally, the molecule's passive diffusion controls the transfer [23]. In cutaneous wound healing, the mechanics are similar as the API enters the bloodstream, but the intended medicinal action is localized. There are two main channels for molecular transfer: transepidermal, which involves a medication passing into cells (intracellular) or across the gaps between cells (transcellular), and transappendageal (by hair, sweat glands, or follicles). An ideal candidate bioactive compound or drug for transdermal drug delivery should possess some physicochemical properties, including molecular weight below 500 Da, high lipophilicity, low-dose therapy, nontoxicity, appropriate water solubility (0.05–1 mg/mL), melting point below 250°C, and a hydrophilic/hydrophobic balance with log P between 1 and 5 [25–27]. Plant-derived antimicrobials have complex molecular structures and poor bioavailability, and their topical delivery is particularly challenging. To circumvent these challenges, new strategies are being explored. These include the use of natural penetration enhancers, such as terpenes, and MN technology, which disrupts the stratum corneum to facilitate delivery [20].

## 3. Classification of Plant-Derived Antimicrobial Agents

**Figure 2:**
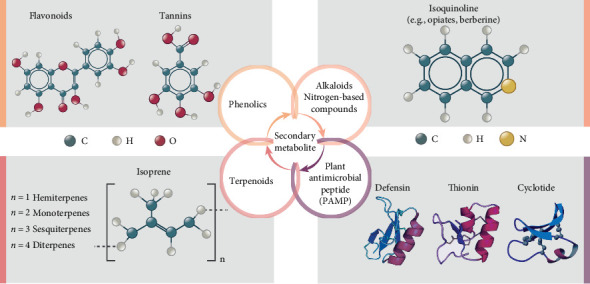
This diagram presents the molecular structures and classifications of major bioactive compounds found in plants, grouped by their chemical nature and biological roles: flavonoids: (a) polyphenolic compounds with a C6-C3-C6 backbone and hydroxyl groups, (b) known for antioxidant, anti-inflammatory, and cardioprotective effects, and (c) function by neutralizing free radicals and regulating cellular processes. Tannins: astringent polyphenols with antioxidant, antimicrobial, and protein-binding properties. Two main types: (a) hydrolyzable tannins (made of gallic/ellagic acid esters) and (b) condensed tannins (flavonoid-based polymers). Alkaloids: (a) nitrogen-containing organic compounds with complex ring structures, (b) exhibit neurological, analgesic, antimicrobial, and anticancer activities, (c) examples: morphine, quinine, caffeine. Plant antimicrobial peptides (PAMPs): (a) small protein molecules part of plant immune defense and (b) act by disrupting microbial membranes or essential processes. Terpenoids (highlighted structure): (a) built from isoprene units (CH_2_-C=CH-CH_2_) and (b) highly diverse with anti-inflammatory, antiviral, and anticancer functions.

There are about 300,000 plant species identified in our world. There are flowering plants, ferns, mosses, and gymnosperms [28]. Of all these, however, only a small number of them are utilized for medicinal purposes, which is about 30,000 globally [29]. The application of using plants as medicine is significantly broader, as plants yield an immense number of new metabolites for protection. So far, most plant chemicals with antimicrobial activity are secondary metabolites, while few have been categorized as primary metabolites. The exact role of secondary metabolites in plants is not known, but it is believed that these compounds play a significant part in the plant's defense against predators and pathogens and also in obtaining advantages in competition for survival [30, 31]. Plant antimicrobial compounds encompass a wide variety of bioactive compounds with diverse chemical structures and pharmacological activities. As can be seen from Figure 2, the phytochemicals possessing antimicrobial activity belong to the following categories:

1. Phenolics and polyphenols
2. Alkaloids
3. Terpenoids
4. Antimicrobial peptides
5. Sulfur-containing compounds

### 3.1. Phenolics and Polyphenols

This is the most widespread class of secondary metabolites, being found universally in the plant kingdom [32]. They are a significant class of secondary metabolites that participate in growth regulation, signaling, and defense and are found in the plant kingdom. This class has a wide range of compounds ranging from simple phenols to phenolic acids, quinones, flavones, flavonoids, and tannins [33]. They are well known for their antimicrobial activity and cell wall and membrane destruction of microorganisms. Flavonoids, for example, are present in fruits, vegetables, and flowers and are well known for their antimicrobial, anti-inflammatory, and antioxidant activity [34, 35]. They inhibit microbial cytoplasmic membrane activity, energy metabolism, and nucleic acid synthesis. Tannins have multiple pharmacological activities, including antioxidant effects and scavenging of free radicals. They are also found to exhibit antimicrobial, anticancer, and cardioprotective activities [36]. Phenolics are all physiologically active compounds having a minimum of one phenolic ring in their molecular structure. Phenolic compounds are end-products of the shikimic and acetic acid biosynthesis pathways. Other than sharing a common origin and structural features, members of this class also resemble one another in pharmacological action, mechanisms, and therapeutic outcomes [37]. Phenolics possess the crucial ability to reduce oxidative tension in living organisms by scavenging free radicals and reactive oxygen and nitrogen species (ROS and RNS) [38], sequestering metallic ions (well known to act as oxidation reaction catalysts) [39], inhibiting principal enzymes (such as lipoxygenases, xanthine oxidase, monoamine oxidase, cyclooxygenases, phosphate oxidase, and nicotinamide adenine dinucleotide) [40, 41], enhancing natural antioxidant defense mechanisms [42], and preventing the formation of ROS/RNS [43]. The antioxidant capacity of phenolics is mostly established by the amount of electron-donating phenolic groups [44]. Phenolic compounds form stable radicals through free radicals and scavenge reactive species, ultimately inhibiting oxidative chain reactions through contact [45]. The potent antioxidant activity of phenolics is responsible for their wide range of health benefits in humans since they play a role in combating oxidative stress, the prime reason behind inflammatory, allergic, carcinogenic, and atherogenic diseases [46, 47]. Various molecular pathways, aside from antioxidant activity, play a role in the diverse health benefits of phenolics, including anti-inflammatory, anticancer, antimicrobial, antiallergic, antiaging/regenerative, immunomodulatory, antiatherogenic, and vasoprotective effects [40, 48]. Other molecular actions, aside from antioxidant effects, are responsible for the multifaceted health effects of phenolics, including anti-inflammatory, anticancer, antimicrobial, antiallergic, antiaging/regenerative, immunomodulatory, antiatherogenic, and vasoprotective effects [49].

### 3.2. Alkaloids

Alkaloids are nitrogen-containing compounds that exhibit a broad spectrum of antimicrobial activities. They can interfere with microbial DNA synthesis, protein production, and other critical cellular processes [50]. Alkaloids are nitrogenous heterocyclic bases with a low molecular weight that are extracted from various sections of plants [51]. They are leftover alkaline compounds resulting from plant metabolism, mostly generated as part of defensive systems. Alkaloids have an extensive range of pharmacological effects and are utilized for medicinal purposes or as recreational substances [52]. Pharmaceutical effects can be classified into various categories, such as stimulants (cocaine), analgesics (morphine), local anesthetics, psychedelics (psilocin), neuro-stimulants (nicotine and caffeine), antibacterial agents (nkolbisine, berberine, and kokusaginine), anticancer medications (vincristine and vinblastine), cholinomimetic (galantamine), antimalarial (quinine), antihypertensive agents (reserpine), vasodilators (vincamine), antiasthma therapeutics (ephedrine), spasmolysis agents (atropine), and antiarrhythmic drugs (quinidine) [53]. Nevertheless, not all of these alkaloids are suitable for use as APIs in transdermal delivery. The medical devices that are already integrated with transdermal patches or those being researched for transdermal therapies are mentioned below.

#### 3.2.1. Opiates (Codeine and Morphine)

Opiates such as codeine and morphine are isoquinoline alkaloids obtained by extracting or refining natural plant material, specifically poppy sap and fibers from *Papaver somniferum* [54]. Morphine is one of the earliest analgesic drugs investigated for tolerance development to minimize the metabolic consequences of ingesting or the pain of intravenous injection [55]. Westerling et al. in 1994 suggested a basic transdermal morphine administration method. The method bypasses the spinal cord by physically extracting it with a vacuum. A lesion forms on the skin and a diffusion chamber with gauze soaked with morphine is placed over it. The diffusion yield reaches 75% within the initial 11 h following application [56]. Despite the limited diffusion rate and therapeutic impact, a transdermal patch with morphine has been created. A transdermal patch composed of polyethylene sponge foam was created to be put directly into the skin as a retention agent. Morphine hydrochloride is administered without the need for invasive permeation enhancers or a membrane that limits the rate of delivery. An adhesive coating is used to affix the patch to a clean, nonhairy area of the skin [57].

#### 3.2.2. Berberine Hydrochloride

Berberine hydrochloride, also known as berberine, is an isoquinoline alkaloid derived from various plants within the *Berberis* genus, such as *Berberis aristata*, *Berberis vulgaris*, and *Berberis aquifolium*, as well as from other genera like *Hydrastis canadensis*, *Coptis chinensis*, and *Phellodendron amurense* [53]. It possesses potent antimicrobial properties, a wide range of antibacterial effects, and promising potential for treating fungal and bacterial infections as well as other skin conditions. Berberine's limited solubility in water and lipids necessitates the use of other chemicals or techniques to aid in transdermal skin delivery [58]. The significance of this alkaloid in treating skin conditions like psoriasis has led to several investigations on effective trans-epidermal transport mechanisms such as nanocarriers and the impermeability of nanobiocellulose with microbial origin [59].

#### 3.2.3. Nicotine

Nicotine is a powerful alkaloid present mainly in Solanaceae family plants. *Nornicotine* is present abundantly in *Nicotiana* species (tobacco) and is also present in 2–7 μg/kg in other edible plants of the Solanaceae family, including potatoes, eggplants of the genus *Solanum*, peppers of the genus *Capsicum*, and *tomatoes* of the genus *Lycopersicon* [53]. There are several commercial nicotine-containing transdermal patches used for the specific indications of smoking cessation or for the treatment of migraine [60]. Either the active ingredient is in a drug reservoir or is incorporated into a matrix. Nicotine is a widely studied and controversial substance due to its potential for addiction and related health risks. While it is most associated with tobacco, it is worth noting that nicotine does occur naturally in numerous plants, particularly those belonging to the Solanaceae family [61]. A number of the most popular consumed vegetables are among these, such as potatoes, eggplants, peppers, and tomatoes. Since these foods contain nicotine, there are concerns about their potential health effects for regular consumers. The use of nicotine-containing transdermal patches for smoking cessation and migraine treatment has become common. The patches deliver controlled levels of nicotine through the skin, providing an alternative to traditional smoking and allowing individuals to wean themselves from the substance [62]. However, the long-term effects and potential risks of using these patches over a long period of time are yet to be studied. Additionally, the ubiquitous presence of nicotine in everyday foods has also provoked concerns about inadvertent ingestion and its implications on overall health. Further research needs to be done to understand the complete implications of nicotine consumption from natural sources and medical therapies. As long as the scientific community is investigating the effect of nicotine on the body, consumers need to be informed of its presence in a variety of products and make informed choices about their use. In addition, health practitioners play a significant role in informing people on the potential benefits and risks of nicotine products and also making access to effective and safe means for the treatment of nicotine dependence and related health diseases [63].

#### 3.2.4. Caffeine

Caffeine is a purine alkaloid present in over 60 plant species worldwide [64]. The alkaloid is a vasodilator and plays a role in numerous nervous system disorders such as epilepsy, migraine, Alzheimer's disease, ischemia, traumatic brain injury, alcoholism, anxiety, and brain cancer [65]. Caffeine can be delivered transdermally through various penetration enhancers or physical approaches such as mechanical, iontophoresis, ultrasound, and MNs to circumvent its limited solubility in the lipid layer of the stratum corneum [25, 66].

#### 3.2.5. Ephedrine

Ephedrine is an alkaloid derived from plants like *Ephedra sinica*, containing a phenethylamine skeleton, which gives the substance its name [67]. Ephedrine induces cardiovascular effects that mirror those of epinephrine, including elevated heart rate, blood pressure, and contractility [68]. Similar to pseudoephedrine, it acts as a bronchodilator but produces a far greater impact. Ephedrine is mostly utilized to diminish the sedative impacts of other motion sickness medications when coupled with a transdermal patch, while it can also help alleviate motion sickness [69].

### 3.3. Terpenoids

Terpenoids represent a vast and diversified group of chemicals that possess strong odors. Terpenoids work either by inhibiting microbial membranes, cell wall formation inhibition, or by interfering with protein function [70]. Terpenoids are modified terpenes where there is movement or loss of methyl groups, or the addition of more functional groups, often oxygen. These two names are frequently used interchangeably [71]. They are found extensively in higher plants, algae, lichens, liverworts, mosses, essential oils, insects, microorganisms, and marine animals [72]. The chemical class is extremely heterogeneous with a wide range of structures and functions. Terpenes and terpenoids are distinguished based on their chemical structure, which consists of many repeated isoprene units (C5H8). Hemiterpenes contain a single isoprene unit, monoterpenes contain two isoprene units (C10), sesquiterpenes contain three isoprene units (C15), and diterpenes contain four isoprene units (C20) [73]. Terpenes are very volatile and susceptible to degradation, primarily by isomerization and oxidation, and are usually thermolabile [74, 75]. Research has focused on delivering hydrophobic terpenoids to specific targets, with applicability across several sectors. These naturally occurring substances have been utilized in transdermal investigations since the 1960s and are known to be an effective and safe class of penetration enhancers [76]. A majority of them have been used as flavoring agents, antispasmodics, carminatives, or perfumes [77]. Several studies have confirmed that terpenoids have the potential to repress nuclear factor-κB (NF-κB) signaling, a key regulator in the development of inflammatory disorders and malignancy, justifying their antineoplastic and anti-inflammatory uses [78, 79]. Terpenes were reported to improve cutaneous wound healing [80]. Due to the immense therapeutic significance of this compound class, it is recognized that appropriate carriers have a crucial role in their successful delivery. Effectiveness, security, and natural delivery techniques are highly sought.

#### 3.3.1. Terpenes as Skin Penetration Enhancers

The success of transdermal delivery of medications relies on the medicines' ability to permeate the skin adequately to achieve the desired therapeutic concentration. The stratum corneum is a significant obstacle to medication absorption via the skin [81]. The stratum corneum is composed of dead cells rich in keratin, enclosed by crystalline lipid lamellar structures in intercellular media [82]. These are uninterrupted formations in the spinal cord that are essential for effective barrier function in the skin. Penetration enhancers are commonly used to boost medication delivery through the skin by temporarily reducing the barrier function of the stratum corneum, thereby increasing percutaneous absorption [83]. Terpenes and terpenoids are highly regarded for their ability to enrich natural goods [84]. They are often regarded as less hazardous and have minimal irritancy potential when compared to other synthesized surfactants or skin penetration enhancers. Furthermore, the Food and Drug Administration (FDA) has classified this group of penetration enhancers as generally recognized as safe (GRAS) substances [85]. Terpenes have the potential to improve skin penetration through several mechanisms, including their interaction with stratum corneum lipids and keratin, as well as their ability to enhance drug solubility in stratum corneum lipids. However, the specific way in which terpenes interact with the stratum corneum in the presence of different solvents may vary due to the unique physical and chemical properties of these solvents and their interactions with the stratum corneum. Nonetheless, specific instrumental methods can be employed to analyze and identify these interactions [85].

#### 3.3.2. Terpenes as Bioactive Compounds

Terpenes and terpenoids are extensively studied for their medicinal potential in topical applications, particularly for their anti-inflammatory properties, such as in healing burns or wounds [86, 87]. Inflammation is the body's natural reaction to tissue harm caused by irritants, stress, radiation, viral and microbial infections, or genetic alterations [88]. It can be acute or chronic. Common indicators of acute inflammation include swelling, discomfort, erythema, and a heightened temperature. Chronic disorders may arise from inflammation, and if the underlying issue is not addressed, the inflammation can progress to subacute or chronic stages. Chronic inflammation has a significant influence on the onset of several diseases, such as cancer, diabetes, cardiovascular disease, asthma, and obesity, in addition to traditional inflammatory conditions like psoriasis or arthritis [89]. Chronic conditions may require the use of anti-inflammatory medications, including steroidal and nonsteroidal pharmaceuticals, which can have adverse side effects [90]. Hence, there is a necessity to discover novel treatment options that are less harmful, with terpenes appearing as ideal possibilities.

### 3.4. Plant Antimicrobial Peptides (PAMPs)

PAMPs are small proteins synthesized by various plants as part of their defense strategy. Most PAMPs are polypeptides, consisting of 10–60 amino acids with a molecular weight between 2 and 13 kDa. These proteins typically carry a positive charge and have a helical structure. The biological effects of PAMPs have been observed against a broad spectrum of microorganisms, although research has primarily focused on their activity against diverse bacterial and fungal species [91]. PAMPs are positively charged peptides that can block ion channels, protein transport, or enzymatic agents, regulate steroid hormones, and interact with RNA and DNA. PAMPs are often used by many species to defend against pests and infections [92]. They exist in many molecular forms, mostly as linear peptides derived from mammals, insects, and plants [93]. Bacteria create polycyclic forms as lantibiotics. On the other hand, different living forms manufacture various circular peptides, including cyclotides by plants, bacteriocins by bacteria, and theta-defensins by mammals [94, 95]. PAMPs have developed distinctively from other antimicrobial peptides because of the existence of cysteine residues, which create multiple disulfide bridges [96]. Cysteine-rich PAMPs can form disulfide bridges that can be stabilized by converting them into cystine-rich peptides (CRPs). PAMPs are components of plants' defensive systems that are extracted from the roots, flowers, leaves, seeds, and stems of different species [97]. PAMPs have features such as positive charge, molecular weight, and amphipathicity with peptides found in insects, microorganisms, and vertebrates [98]. These qualities pertain to their protective functions. PAMPs have distinctive characteristics, such as a molecular weight between 2 and 6 kDa and containing two or six intramolecular disulfide bonds. They are classified based on conserved secondary and tertiary structures, sequence similarity, and the presence of cysteine motifs. PAMPs possess a compacted structure and are derived from ribosomes with three domains: a mature PAMP domain and N- and C-terminal pro-domains [99]. This process entails the production and storage of peptides from plant organs and reproductive tissues to serve as the primary defensive mechanism against attacks [100]. PAMPs can create ion channels that lead to the release of ions like K^+^ and other cellular components, resulting in the suppression of pathogen cell proliferation and cell demise [101]. PAMPs are considered promiscuous since they exhibit many activities despite having the same structure. Various mechanistic models, such as the barrel stave model, the toroidal pore model, and the carpet model, have been suggested to elucidate the many features displayed by PAMPs [102].

PAMPs have opened up new possibilities in biotechnology for medication discovery for treating human infections and disorders, ranging from systemic to topical applications [103]. Studying the chemical composition and alterations of certain peptide residues might improve the bioactivity of PAMPs. This research could also aid in the discovery of novel peptides in host plants for use as biocontrol agents or in healthcare applications. Environmentally friendly and effective methods can be used to enhance recycling for sustainable development and convert used PAMPs into high-value antimicrobial products. Keratinolysis can be utilized for recycling keratin peptides, with the resulting hydrolyzate showing the ability to inhibit *Escherichia coli* growth [104]. Bioactive peptides are vital elements of plants' defense systems, playing a crucial role in providing rapid protection against bacterial and fungal diseases [105]. PAMPs have antimicrobial properties and participate in cellular signaling [106]. Various active peptides have been collected and separated from different parts of plants and categorized according to their amino acid sequence, location, and the number of cysteine residues contributing to disulfide bridge formation [107]. Ten families of PAMPs have been identified, with thionins, defensins, and snakins being the most extensively researched groups [108]. The initial plant-based antimicrobial peptide is purothionin, which has efficacy against *Pseudomonas solanacearum*, *Corynebacterium fascians*, and *Corynebacterium poinsettia* [109]. Plant defensins are cysteine-rich peptides with four disulfide bridges and a globular form. They are basic peptides consisting of 45–54 amino acid residues, often found across the plant kingdom, and have antimicrobial activity against bacteria and fungi. PvD1 peptide, a defensin found in *Phaseolus vulgaris*, hinders the growth of yeasts like *Candida tropicalis*, *Saccharomyces cerevisiae,* and *Candida albicans* [110].

Thionins are basic peptides consisting of 45–47 amino acids that are present in many plant tissues and have toxic effects on phytopathogenic fungi and bacteria [111]. Snakins are short peptides containing 12 cysteine residues that create six disulfide bridges, which are crucial for their biological function [112, 113]. Snakin-Z, derived from *Ziziphus jujuba*, consists of 31 amino acids and has greater toxicity toward fungi compared to bacteria [113]. Various PAMPs have been found in avocado and capsicum fruits. These PAMPs possess antimicrobial capabilities that might potentially be utilized in treating illnesses caused by *Staphylococcus aureus* and *Escherichia coli* bacteria [114, 115]. PAMPs, due to their effectiveness and wide-ranging action, might be a favorable substitute for traditional antibiotics in fighting infections. The clinical use of PAMPs is restricted due to their limited metabolic stability, which is commonly viewed as an inherent danger of medicinal peptides. Peptides have low oral bioavailability due to their vulnerability to metabolic processes and limited capacity to pass through the intestinal mucosa, which hinders their suitability for oral administration [116, 117]. Moreover, the systemic delivery of peptides, such as by intravenous injection, is restricted by a short half-life due to rapid breakdown by enzymes in the blood and increased elimination by the liver and kidneys [116]. Local application of PAMPs is the most widely used and successful method for delivering peptides to the skin, particularly to wounds, since it allows for targeted distribution and increased peptide concentrations at the intended site [118]. Even with targeted administration, the peptides are susceptible to degradation by many elements in the wound microenvironment. An improved topical formulation of PAMPs is necessary to tackle stability concerns and enhance wound healing benefits.

### 3.5. Sulfur-Containing Compounds

Plants can provide sulfur-containing chemicals with various biological effects as antioxidant, anti-inflammatory, and anticancer agents [119]. Organosulfur natural products (ONPs) are organic compounds containing sulfur components like thioesters, sulfoxides, and thiols that are significant in the pharmaceutical sector [120, 121]. Studies mostly examine S-alk(en)yl cysteine sulfoxides (ASCOs) and glucosinolates, although other sulfur-containing secondary metabolites from plants such as SO_2_, H_2_S, and antimicrobial peptides are also of interest for investigation [122]. Glucosinolates are sulfur-containing phytoanticipins present in Brassicaceae plants like cabbage, broccoli, and cauliflower. ASCOs are mainly found in *Allium* vegetables such as garlic, onion, and leek, and their thiosulfinated derivatives contribute to the flavor compounds of these vegetables [123]. Organic sulfur-containing substances are utilized in topical drug delivery systems together with other pharmacological agents in lipid-based nanocarriers for treating wounds and psoriasis, and they exhibit antitumoral or antibacterial effects [124].

## 4. Mechanism of Action for Plant-Derived Antimicrobial Agents

**Figure 3:**
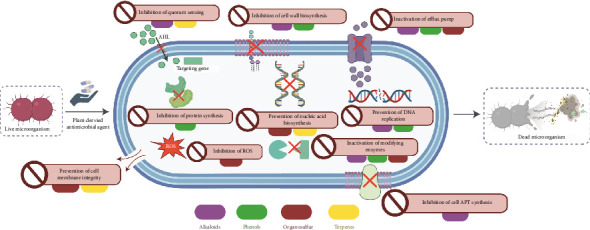
This diagram shows how plant-based antimicrobial compounds inhibit or kill microorganisms by targeting multiple essential microbial structures and processes: (a) Cell wall disruption: some compounds break down or weaken microbial cell walls, causing cell lysis. (b) Inhibition of cell membrane biosynthesis: certain agents disrupt the formation and function of cell membranes, leading to instability and cell death. (c) Inhibition of protein synthesis: some plant compounds block ribosomes, preventing microbes from making vital proteins and halting their growth. (d) Inhibition of nucleic acid synthesis: certain extracts interfere with DNA or RNA replication, stopping microbial reproduction. (e) Modulation of reactive oxygen species (ROS): plant compounds can act as antioxidants or induce oxidative stress in microbes, damaging or killing them. (f) Efflux pump inhibition: some agents block bacterial efflux pumps, increasing the effectiveness of antimicrobial compounds and helping overcome drug resistance. (g) Quorum sensing inhibition: certain extracts disrupt bacterial communication systems, reducing virulence, biofilm formation, and resistance.

As shown in Figure 3, plant-derived antimicrobial agents combat microbial infections at the skin level through various mechanisms.

### 4.1. Disruption of Cell Membrane Integrity

Many of these compounds, such as terpenoids and phenolics, can integrate into the lipid bilayer of microbial cell membranes, causing increased permeability and eventual cell lysis [125]. Phenolic substances such as gallic acid, epicatechin gallate, epigallocatechin gallate, and caffeic acid have been shown in multiple investigations to disrupt the integrity of the bacterial cell wall, resulting in structural damage and leaking of cellular components [126, 127]. The active phenolic compounds bind with the peptidoglycan layer by covalent and/or hydrogen bonds in Gram-positive bacteria and with lipopolysaccharides in Gram-negative bacteria [128, 129]. Additionally, phenolics exhibit inhibitory effects on the penicillinase enzyme as well as the efflux pump, leading to reduced antibiotic resistance and enhanced antibacterial efficacy when combined with antibiotics [130]. Many phenolic compounds are known to have antibacterial properties by modifying the fluidity, permeability, ionic transportation, and respiration of bacterial cell membranes [131]. The phenolic molecule's chemical structure, including its molecular mass, polarity, and conformation, along with its placement within the lipid bilayer, can lead to either fluidization or rigidification [132]. Flavonoids like chrysin, kaempferol, baicalein, quercetin, epigallocatechin gallate, luteolin, theaflavin, gallocatechin, and theaflavin gallate reduce bacterial cell membrane fluidity, whereas isoflavonoids such as ononin, puerarin, genistein, daidzein, and stilbene resveratrol exhibit the opposite effect [133, 134]. Phenolics reacting with enzymes important for the stability of cell membranes can lead to the destabilization of bacterial cell membranes [129]. Furthermore, some phenolic acids, such as gallic and caffeic acids, can cause acidification of the bacterial membrane, resulting in its breakdown and altering ion transport and permeability. Galangin, a flavonoid included in propolis, has been discovered to cause membrane disruption and loss of potassium from the intercellular space in bacteria, contributing partially to the antibacterial activities of propolis [135, 136].

### 4.2. Inhibition of Cell Wall Synthesis

Some plant compounds interfere with the synthesis of microbial cell walls, weakening them and causing cell lysis. Peptidoglycan is an essential component of the bacterial cell wall, and the inhibition of its synthesis is a common mechanism of action for plant-derived antimicrobials. Certain plant-derived compounds, like flavonoids, have been shown to inhibit enzymes involved in peptidoglycan biosynthesis, such as D-alanine: D-alanine ligase [136]. This enzyme is responsible for producing the terminal dipeptide of the peptidoglycan precursor. By binding to and inhibiting these enzymes, the plant compounds disrupt the synthesis of the bacterial cell wall, leading to cell death. Other plant antimicrobials, like catechins from green tea, have been found to interfere with the biosynthesis of the bacterial cell wall by binding directly to the peptidoglycan layer. The inhibition of cell wall synthesis is particularly effective against Gram-positive bacteria, whose cell walls are primarily composed of peptidoglycan [127].

### 4.3. Inhibition of Protein Synthesis

Microbial protein synthesis is a common target for natural antimicrobial compounds from plants. Plant-derived antimicrobials can interfere with various stages of the microbial protein synthesis process. Some plant compounds bind to the 30S or 50S ribosomal subunits in bacteria, preventing the assembly of the functional 70S ribosome. This blocks the initiation, elongation, or termination steps of protein synthesis [137]. Besides, certain plant antimicrobials can prevent tRNAs from properly binding to the ribosomal A, P, or E sites. This inhibits the delivery of amino acids to the growing polypeptide chain [136].

### 4.4. Interference of Enzyme Activity

Interference of enzyme activity is a mechanism by which plant-derived agents can exhibit antimicrobial properties. Many plant-derived compounds have been found to inhibit the activity of enzymes for microbial growth. The inhibition of enzyme activity can occur through various mechanisms, including binding to the enzyme's active site, preventing substrate access and catalysis, altering the enzyme's structure (leading to conformational changes and reduced activity), interfering with cofactor binding (which is necessary for enzyme function), and inducing oxidative stress, which can damage enzymes and impair their activity. The specific enzymes targeted by plant-derived agents vary depending on the microorganism and the compound involved [138–140].

### 4.5. Interference With Nucleic Acid Synthesis

Alkaloids and some flavonoids can bind to microbial DNA or RNA, inhibiting nucleic acid synthesis and thus preventing cell replication and function [70]. Typically, the physiologically active aglycons of phenolic compounds have a structure that is abundant in reactive functional groups, numerous phenolic groups, carbonyl groups (such as most flavonoids, anthraquinones, and xanthones), and free or esterified carboxylic groups [37]. They readily engage in hydrogen bonding with various biomolecules such as proteins (including receptors and adhesins), nucleotides, enzymes like topoisomerases, proteases, and transcriptases [141, 142]. They also form complexes with essential metallic ions (such as iron ions) crucial for the infectious cycle of pathogenic microbes, facilitating processes like entry, replication, adhesion, and spreading [143, 144]. Researchers have identified broad and nonspecific results of possible interactions among phenolics that contribute to their antibacterial and antiviral activities. Curcumin demonstrates antiviral activity against human herpesvirus-1 and other DNA viruses by inhibiting the histone-acetyltransferase activity associated with particular transcriptional coactivator proteins, as evidenced by examples related to skin infections [145]. Curcumin has been discovered to prevent the adhesion and reproduction of human herpesviruses 2 and 1 by inhibiting adhesins [146]. Epigallocatechin gallate exhibits antibacterial activity against methicillin-resistant *Staphylococcus aureus* (MRSA), not only by damaging bacterial cell walls but also by blocking multiple *staphylococcal virulence* factors [134]. Kaempferol, quercetin, and other flavonoids hinder the activity of *staphylococcal* topoisomerases [147]. Phenolic chemicals are now widely accepted to possess antiviral, antibacterial, and antifungal properties following years of research [148]. They have demonstrated efficacy against common pathogens responsible for skin infections, including *Enterococcus* bacteria, *Staphylococcus*, *Herpes* virus 1 and 2, *Pseudomonas*, and dermatophytes like *Epidermophyton*, *Trichophyton*, and *Microsporum* species [149, 150]. Thus, using them topically is very advantageous for treating infectious skin conditions such as infected wounds, herpes infections, impetigo, dermatophytosis, and others.

Dermal treatments containing phenolic compounds are receiving special attention for treating antibiotic-resistant infections, which are more widespread [151]. The precise mechanism by which a certain phenolic molecule exhibits antibacterial action is not always widely researched and completely understood. The importance of several additional phenolic compounds as antibacterial agents, in addition to those mentioned before, should be recognized. The primary active components in *Hypericum perforatum* preparations include pseudohypericin, hypericin, resveratrol, hyperforin, vitexin, hesperidin, isovitexin, and eugenol [152, 153].

### 4.6. Antioxidant Activity

The biological activity and concentration of active ingredients in plants are mainly influenced by the growing environment, harvest time, and conditions during the drying and storage of the herbal material [154, 155]. Many plant-derived compounds, such as polyphenols, flavonoids, and terpenoids, have the ability to act as antioxidants or pro-oxidants, depending on the environment. In the context of antimicrobial activity, certain plant antimicrobials can paradoxically function as pro-oxidants, generating ROS within microbial cells. ROS include free radicals like superoxide anion (O_2_•), hydroxyl radical (•OH), and nonradical species like hydrogen peroxide (H_2_O_2_). ROS can cause severe oxidative damage to microbial cellular components, including lipid peroxidation of cell membranes, oxidation of proteins (leading to enzyme inactivation and structural changes), and DNA damage (causing mutations and disrupting replication). The oxidative stress induced by ROS ultimately leads to microbial cell death through apoptosis-like programmed cell death pathways. The antioxidant activity of plant antimicrobials is particularly effective against drug-resistant microbial strains as it targets fundamental cellular processes [156, 157].

### 4.7. Inactivation of Efflux Pumps

Inactivation of efflux pumps is a key mechanism by which some plant-derived agents can exhibit antimicrobial activity and potentiate the effects of antibiotics [158–160]. Efflux pumps are membrane transport proteins that actively extrude antimicrobial agents and antibiotics out of bacterial cells, conferring resistance [160]. By inhibiting or inactivating these efflux pumps, plant-derived compounds can:

1. Increase the intracellular concentration of antibiotics, making bacteria more susceptible.
2. Prevent the extrusion of plant-derived antimicrobial compounds, enhancing their potency.
3. Disrupt biofilm formation, which is associated with increased efflux pump activity [159–161].

By blocking efflux pumps, these plant compounds can potentiate the activity of antibiotics and restore susceptibility to resistant bacteria. Inactivation of efflux pumps is a promising approach to combat multidrug resistance and enhance the efficacy of antimicrobial agents [161].

### 4.8. Quorum Sensing (QS) Inhibition

QS is a cell-to-cell communication system used by many bacteria to coordinate group behaviors, including the expression of virulence factors and biofilm formation [162]. QS relies on the production, detection, and response of small signaling molecules called autoinducers. As bacterial cell density increases, the autoinducer concentration rises, triggering changes in gene expression. Certain plant-derived antimicrobial compounds have been shown to interfere with bacterial QS systems, disrupting their ability to coordinate virulence and survival strategies. The mechanisms of QS inhibition by plant antimicrobials include inhibition of autoinducer synthesis, interference with autoinducer detection, degradation of autoinducers, and inhibition of QS-regulated gene expression [163]. By targeting bacterial QS, plant-derived antimicrobials can attenuate virulence without killing the bacteria outright. This reduces selective pressure for developing antibiotic resistance. QS inhibition has been demonstrated against a wide range of pathogenic bacteria, including *Pseudomonas aeruginosa*, *Staphylococcus aureus*, and *Vibrio* species [164]. According to the mentioned mechanisms, many plant extracts likely act through a combination of these mechanisms (targeting multiple sites), making it more difficult for microorganisms to develop resistance.

## 5. Conventional Topical Delivery Methods

Two methods exist for administering drugs through the skin: dermal and transdermal. Dermal administration aims to confine the drug within the skin's layers, avoiding systemic absorption as much as possible. This approach reduces or eliminates the risk of systemic side effects. On the other hand, transdermal administration involves transporting the drug through the skin's dermis to enter the systemic circulation, utilizing carrier systems. The benefits of transdermal delivery include improved patient adherence due to the ease of treatment, the option to stop treatment when necessary, controlled release of medication ensuring steady levels in the bloodstream, and bypassing the liver's first-pass metabolism, which can degrade drugs before they circulate throughout the body [165]. Traditional dermal delivery methods, such as creams, ointments, and lotions, have long been employed for the topical administration of antimicrobial agents [166]. These formulations are designed to provide localized treatment of skin conditions, offering the advantages of ease of use and minimal systemic side effects. Creams are emulsions that typically offer a balance between oil and water, making them less greasy and more cosmetically acceptable than ointments [167]. They are suitable for moist or weeping skin conditions. Ointments are semisolid preparations that are more occlusive and greasier than creams, providing a barrier that enhances the skin's hydration [167]. They are best suited for dry and flaky skin conditions. Lotions are liquid emulsions that are lighter and less viscous than creams or ointments, making them easier to apply over larger areas of the skin. Lotions are particularly useful for conditions where a cooling effect is desired. Despite their widespread use, these conventional methods have limitations, including poor penetration of the active ingredients through the skin barrier, the potential for irritation and sensitization, and the instability of some compounds in the formulation [168].

## 6. Advanced Topical Delivery Systems: Lipid-Based Topical Delivery Systems

In order to overcome the limitations of traditional formulations, newer topical drug delivery systems have been developed to increase their stability and antimicrobial activity, provide sustained release patterns, and improve their penetration and bioavailability [16]. The newer delivery systems reduce the limitations of traditional formulations by improving the bioavailability of antimicrobial compounds, giving controlled release profiles, and decreasing side effects [169]. Their development is a landmark toward the treatment of skin infections, enabling more effective and patient-friendly treatments. With ongoing advances in research, these technologies also continue to enhance with potential for further improvement in dermal drug delivery. Some natural and synthetic polymers are well established to be beneficial for drug delivery through numerous routes of administration. Their applicability rests in some key characteristics such as safety, biodegradability, extended and controlled drug release, and improvement in drug absorption and efficacy. All these characteristics render them invaluable when it comes to delivering drugs in a manner that they are performed with optimal efficiency and less side effect [170, 171].

### 6.1. Lipid-Based Topical Delivery Systems

#### 6.1.1. Lipid NPs

Delivery systems like solid lipid NPs and polymeric NPs for NP delivery can enhance the stability and penetration of the entrapped antimicrobial agents, into the skin [9, 172]. Small-sized NPs are able to penetrate the stratum corneum more easily, carrying the active ingredient deeper into the skin layers or even systemically. In recent years, SLNs, NLCs, and lipid nanocapsules (LNCs) have been described as lipid-based carriers because of their advantageous characteristics [173, 174]. SLNs are prepared by replacing the liquid lipid in oil-in-water (O/W) nanoemulsions with solid lipids, such as beeswax, carnauba wax, triglycerides, acetyl alcohol, and cholesterol butyrate. When dispersed in water, the lipids solidify at room temperature and are stabilized by surfactants [175]. In wound healing, EO-loaded lipid NPs have been extensively used as effective antibacterial alternatives, aiming at microbial colonization for antibiotic-free wound healing [176]. Saporito et al. formulated SLNs and NLCs with either eucalyptus or rosemary EOs [176]. These formulations were made using cocoa butter, olive oil, or sesame oil as the solid and liquid lipids and pullulan to increase solution viscosity. They reported the physicochemical properties, biocompatibility, growth against normal human dermal fibroblasts, and antimicrobial activity of NPs against two Gram-positive bacteria (*Staphylococcus aureus* and *Streptococcus pyogenes*). The potential of the NPs to heal wounds in vivo was then tested on a rat burn model. Studies indicated that the resulting NLCs were stable, bioadhesive, and flexible, which could enhance interaction with the biologic substrate and the subsequent bioadhesive joint formation. Moreover, NLC showed excellent biocompatibility and proliferation features toward fibroblasts. They promoted increased in vitro cell proliferation (*p* < 0.05) and exhibited antimicrobial activity against Gram-positive bacteria, along with enhancing wound healing through re-epithelialization and the formation of the stratum corneum. The authors attributed these promising therapeutic effects to the synergistic action of *olive* and *eucalyptus* EO [176]. Other scientists have also tried to integrate NLCs with gels to simplify the topical formulation. In such a study, Ghodrati et al. have developed a xanthan gel loaded with peppermint oil-loaded NLCs, where Precirol® ATO 5 was used as the solid lipid and Miglyol 812® as the liquid lipid. The group performed extensive in vitro characterization and performed the antibacterial activity using a microdilution assay. They also performed in vivo studies in infected wound healing in mice, such as histopathological examination, granulation tissue examination for total bacterial colony count, and wound immunofluorescent staining to measure fibroblast gene expression profiles. The *peppermint* EO was encapsulated effectively into the lipid matrices with 90% encapsulation efficiency and proved to be a potent antibacterial agent in free and encapsulated state against some pathogens like *Staphylococcus epidermidis*, *Staphylococcus aureus*, *Listeria monocytogenes*, *Escherichia coli*, and *Pseudomonas aeruginosa*. The therapy failed against *Bacillus anthracis*, *Salmonella typhimurium*, and *Streptococcus pneumoniae*. The in vivo studies indicated that the NLCs enhanced wound healing by reducing wound area and edema, lowering the total bacterial count, and accelerating neovascularization, fibroblast infiltration, and re-epithelialization [177]. Khezri et al. also ventured into the development of topical gels incorporating rosemary-loaded NLCs aimed at treating infectious wounds [178]. These NLCs were formulated using Precirol® as the solid lipid and Miglyol® as the liquid lipid. They investigated the antibacterial effect of NLCs and the resultant gels by the disc diffusion procedure. Both the NLCs and the resultant gels demonstrated efficacy in decreasing the counts of various bacterial strains, including *Staphylococcus epidermidis*, *Staphylococcus aureus*, *Listeria monocytogenes*, *Escherichia coli*, and *Pseudomonas aeruginosa* (*p* > 0.05). After inducing infection and applying dressings in the animal model, histological evaluation, and measured tissue bacterial counts, along with serum levels of SDF-1α, IL-3, IL-10, and VEGF were performed. The designed formulation showed significant potential for promoting wound healing. This was evidenced by the reduction in tissue bacterial colonization rates (*p* < 0.05) and the enhancement of several key healing processes, such as neovascularization (*p* < 0.05), re-epithelialization, wound contraction, and the levels of serum inflammatory cytokines (*p* < 0.001) [178].

**Figure 4:**
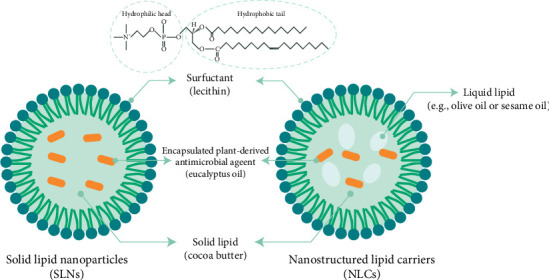
The diagram highlights the structural differences between two types of solid lipid nanoparticle (SLN) drug delivery systems: SLNs and nanostructured lipid carriers (NLCs). Both systems encapsulate therapeutic agents to improve stability, bioavailability, and controlled drug release. SLNs consist of a solid lipid matrix that remains solid at body temperature, with the drug either embedded in the core, adsorbed on the surface, or dispersed within the matrix. While SLNs provide prolonged release, enhanced stability, and reduced toxicity, their highly ordered crystalline structure limits drug-loading capacity. In contrast, NLCs are an advanced version, combining solid and liquid lipids to create a less-ordered matrix, which increases drug-loading capacity and prevents drug leakage during storage. NLCs offer superior stability, higher drug entrapment efficiency, and more controlled release, making them suitable for pharmaceutical and cosmetic applications. Both SLNs and NLCs are used in targeted drug delivery, transdermal systems, and oral or parenteral administration, offering benefits such as biocompatibility, improved solubility for poorly water-soluble drugs, and the potential for ligand-based functionalization for targeted therapy.

The same researchers explored the use of another EO, *Mentha pulegium* L. (pennyroyal), for treating infectious wounds by creating NLCs and their corresponding gels. They investigated the in vitro antibacterial activity of pennyroyal-loaded NLCs and in vivo wound healing activity of them in the mice model. To assess the effect of pennyroyal-loaded nanostructures on wound healing, tissue bacterial count, and wound contraction, molecular and histological parameters were evaluated at 3, 7, and 14 days after forming wound. Similarly to their findings with rosemary-loaded NLCs, the pennyroyal-loaded systems demonstrated enhanced antibacterial activity against *Staphylococcus epidermidis*, *Staphylococcus aureus*, *Listeria monocytogenes*, *Escherichia coli*, and *Pseudomonas aeruginosa* (*p* < 0.01). These formulations also promoted wound healing by stimulating cell proliferation. Moreover, they were found to effectively reduce inflammation by upregulating the gene expression of key anti-inflammatory and healing-promoting factors, such as TGF-β, IL-10, and b-FGF (*p* < 0.05), showcasing the broad therapeutic potential of EO-loaded NLC systems in wound care [179]. A typical structure of a SLN with EO is shown in Figure 4.

#### 6.1.2. Emulsion-Based Systems

Nanoemulsion, or microemulsion, is a type of colloidal nanosystem made up of a blend of oil, water, and an appropriate stabilizing surfactant [9, 180]. Nanoemulsions are composed of tiny droplets in the nanometer range that are stabilized by emulsifiers. Nanoemulsions are recognized as a promising method for delivering antimicrobials because of their inherent stability, antimicrobial abilities, capacity to enhance drug solubility, cellular and organ targeting features, capacity to target biofilms, and potential to combat AMR. Nanoemulsions can be delivered by several methods, including dermal, transdermal, oral, pulmonary, parenteral, nasal, ophthalmic, and rectal [181–183].

**Figure 5:**
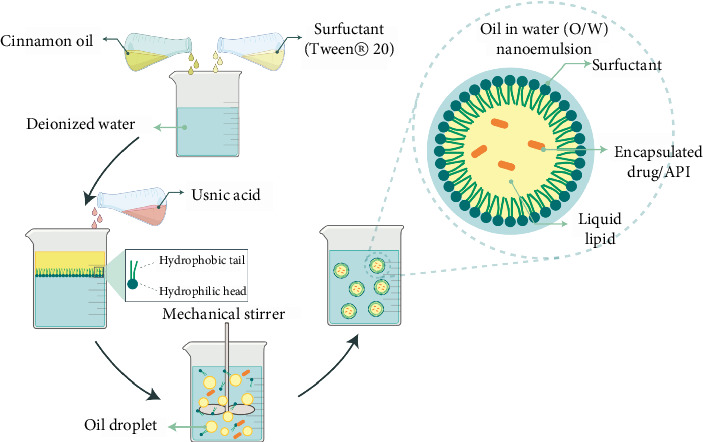
Demonstrates the preparation of an emulsion-based carrier system, using cinnamon oil and water to form an oil-in-water (O/W) emulsion. The process begins by pouring cinnamon oil into water, where surfactant molecules stabilize the mixture by reducing interfacial tension—their hydrophilic heads interact with water while their hydrophobic tails bind to the oil, preventing droplet coalescence. Shear forces are then applied to break the oil into nanosized droplets, creating a stable, homogenous nanoemulsion invisible to the naked eye. Finally, the emulsion is filtered to eliminate any remaining large droplets, ensuring uniform particle size distribution for enhanced stability and efficiency. This method is crucial for developing consistent and effective emulsion-based delivery systems.

Many plant-derived compounds have poor water solubility, limiting their absorption through the skin. Techniques such as the use of solubilizers, cosolvents, or the formation of microemulsions can enhance the solubility of these compounds, improving their dermal delivery [184]. Kumur et al. developed a nanoemulsion that combined *cinnamon* oil and usnic acid to explore its antifungal properties (Figure 5). This nanoemulsion consisted of *cinnamon* oil as the EO, Tween® 20 as the surfactant, ethanol as the cosurfactant, usnic acid as the API, and deionized water as the base of the continuous phase. The effectiveness of this formulation was tested against *Candida albicans* and *Trichophyton mentagrophytes* using two animal models: rats with cutaneous candidiasis and guinea pigs with dermatophytosis, respectively. Topical use of nanoemulsion on rats revealed significant efficiency against cutaneous candidiasis compared to control group animals. In the group treated with the *cinnamon* EO and usnic acid nanoemulsion, only one out of six animals presented a positive fungal culture. In contrast, in the groups treated with the drug solution and the control group, four and six animals, respectively, showed positive cultures. The group receiving the nanoemulsion treatment exhibited a significant decrease in the number of colony-forming units (CFUs) compared to both the untreated control group and the group treated with the drug solution alone. On the other hand, the clinical efficacy profile of nanoemulsion on dermatophytosis guinea pig model was more than the standard drug (ciclopirox) (41.3% and 38.0%, respectively) and drug solution (35.6%). Similarly, mycological efficacy for all treatment groups was considerably better than that for the infected untreated control group. Moreover, the mycological efficiency of nanoemulsion (81.4%) was more than that of the other treated groups (drug solution, 74.5%, and ciclopirox, 79.6%). These results suggest the nanoemulsion's potential as an effective topical antifungal preparation [185].

Plant-based antimicrobial agents are light, temperature, or pH-sensitive and susceptible to degradation. Antioxidants, chelating agents, or encapsulation can protect such labile compounds from degradation, enhance their shelf life, and preserve their activity. Encapsulation of EO in emulsion-based systems, such as microemulsions and nanoemulsions, is considered a strategic approach to enhance the physical stability of these bioactive compounds [186]. It protects the EOs from environmental agents like oxidation, light, and high temperature, reducing their volatility, prolonging the release duration of the EOs, and increasing their bioactivity.

In a further effort to enhance nanoemulsion stability, Bonferoni et al. investigated the effective use of chitosan (CS) oleate as an amphiphilic stabilizer in creating a lemongrass oil (*Cymbopogon citratus*) nanoemulsion. Notably, CS oleate results from the ionic interaction between CS and oleic acid, both known for their antimicrobial properties, potentially augmenting the EO's antimicrobial efficacy. The study demonstrated the development of a stable, small, and monodispersed nanoemulsion, because of the creation of a CS shell upon the oil droplets, which is indicated by a positive zeta potential. This CS shell also renders the nanoemulsion mucoadhesive. The antifungal efficacy and antimicrobial efficacy of CS oleate salt and *lemongrass* EO nanoemulsions against 10 fungal isolates and nine bacterial isolates, all related to a clinical setting, were tested by them. Some of them are involved in vaginal or ophthalmic infections, where the CS mucoadhesive properties can enhance residence time and thus the efficiency of the preparation. Cytotoxicity test was conducted on four various cell lines (Caco-2, HEp-2, McCoy, and WKD cells). Results indicate that the *lemongrass* EO nanoemulsion was biocompatible with various human epithelial cell lines and had enhanced antibacterial and antifungal activity against several pathogens causing ocular and vaginal infections. This suggests its potential application in treating mucosal and/or skin lesions topically [187]. Moghimi and colleagues assessed the antibacterial efficacy of *Thymus daenensis* EOs in both pure and nanoemulsion states against *Escherichia coli* [188]. Converting the EOs (*T. daenensis*) into a nanoemulsion significantly boosted its antibacterial effectiveness against *Escherichia coli*, a significant food-borne pathogen.

The researchers suggest that the antibacterial nanoemulsion's method of action against *Escherichia coli* includes getting the EO near the cell membrane. Hydrophobic compounds in EOs can disrupt cell membranes by affecting the phospholipid bilayer's integrity or interfering with active transporters inside the bilayer. Alterations in the permeability of the damaged cell membrane lead to the leakage of proteins, nucleic acids, and potassium from the cell, causing cell death within 5 min. Nanoemulsions are highly effective delivery technologies for EOs because they may enhance the application of antimicrobials and improve their effectiveness. Hassanshahian et al. obtained the EOs from the tropical *Alhagi maurorum* plant and created a nanoemulsion utilizing the ionotropic gelation process using CS as a nanocarrier [189]. An assessment was conducted to determine the impact of a nanoemulsion on the antibacterial, plasmid curing, and antibiofilm properties of six antibiotic-resistant pathogenic bacteria, namely, *Escherichia coli, Pseudomonas aeruginosa, Staphylococcus aureus, Acinetobacter baumannii, Bacillus cereus*, and *Klebsiella pneumoniae*. The nanoemulsion exhibited antibacterial activity, including antibiofilm properties, and demonstrated superior suppression of bacteria in comparison to the free EO. Statistical analysis also confirmed that the inhibitory effect of nanoemulsion against bacterial biofilm was significant (*p* < 0.05). Zhao et al. developed a self-nano emulsifying medication delivery system including buckwheat flavonoids using a pseudo-ternary phase diagram [190].

The antibacterial efficacy of the nanoemulsion and suspension of flavonoids was tested against *Staphylococcus aureus*, *Escherichia coli*, and *Candida albicans*. The minimum inhibitory concentration (MIC) and minimum bactericidal concentration (MBC) results showed that the antimicrobial activity of suspensions and nanoemulsions increased with higher drug concentrations. The nanoemulsion demonstrated significantly greater activity than the suspension against the three bacteria. Nanoemulsions are very efficient delivery methods for antibacterial agents because they may enhance the application of antimicrobials and improve their effectiveness, as well as dermal and transdermal drug delivery [190].

##### 6.1.2.1. Nanoemulsions' Antimicrobial Mechanisms

1. Mechanisms in EO-in-water (O/W) nanoemulsions
  a. Targeting Gram-positive bacteria: O/W nanoemulsions, such as those containing *Thymus vulgaris* EO, disrupt the peptidoglycan layer of Gram-positive bacteria. The hydrophobic components of the oil phase integrate into the bacterial membrane, causing increased permeability and cell lysis [125, 191].
  b. Targeting Gram-negative bacteria: The lipopolysaccharide outer membrane of Gram-negative bacteria presents a challenge for O/W nanoemulsions. However, the addition of surfactants can enhance penetration by destabilizing the lipopolysaccharide layer, allowing the active compounds to reach the cytoplasmic membrane [191, 192].
2. Mechanisms in water-in-oil (W/O) nanoemulsions
  a. Targeting fungi: W/O nanoemulsions, such as those containing *Cinnamomum zeylanicum* EO, are particularly effective against microbial pathogens. The oil phase disrupts the microbial cell membrane, while the water phase delivers hydrophilic compounds and subsequently can cause microbial death [191, 193].
  b. Targeting biofilms: W/O nanoemulsions can penetrate biofilms formed by bacterial and fungal pathogens. The oil phase disrupts the extracellular polymeric matrix, while the water phase delivers antimicrobial agents that kill the embedded microorganism [182].
3. Mechanisms in microemulsions
  a. Broad-spectrum activity: Microemulsions, with their ultra-small droplet size and high stability, exhibit broad-spectrum antimicrobial activity. For example, microemulsions containing *Mentha piperita* EO have been shown to inhibit bacteria and fungi by disrupting membrane integrity and inhibiting key enzymes [194–196].
  b. Enhanced penetration: The small droplet size of microemulsions allows for enhanced penetration into the skin and microbial cells, making them effective for treating deep-seated infections [196, 197].

#### 6.1.3. Liposomes

Liposomes are among the most widely used colloidal drug delivery systems, having garnered significant attention in both the cosmetics and pharmaceutical industries [198, 199]. Liposomes are small, spherical structures composed of one or more phospholipid bilayers encapsulating aqueous compartments or units. Liposomes stand out among NPs because of their unique capability to encapsulate hydrophilic drugs within their aqueous compartments and hydrophobic drugs within the lipid bilayer. This versatility significantly broadens the range of drugs that can be incorporated into their structure [200].

**Figure 6:**
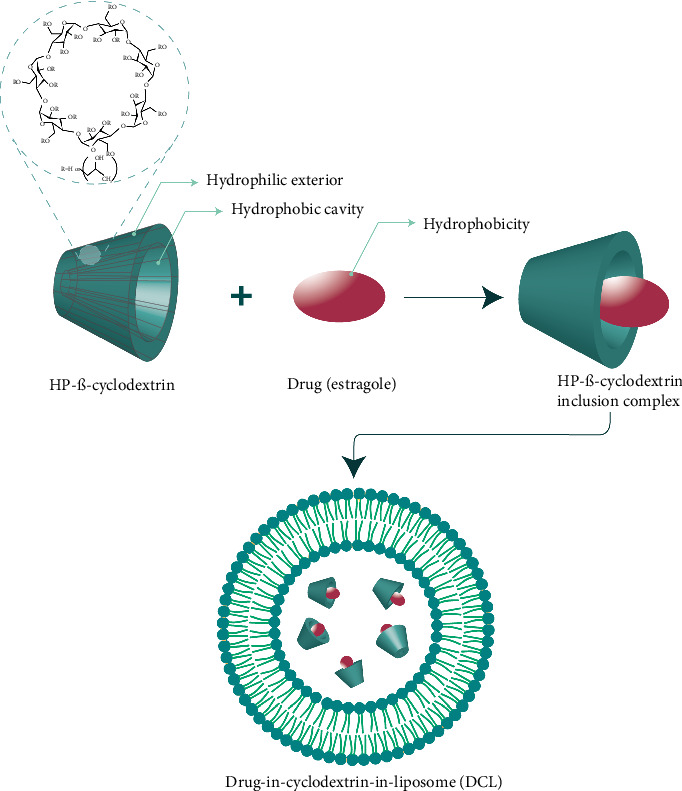
Structure of a carrier-based liposome. This figure illustrates the structure of a novel drug delivery system. This carrier system is designed to enhance the delivery of therapeutic agents, particularly those that are hydrophobic. The outermost layer of the DCL is hydrophilic. It allows the DCL to circulate freely within the watery environment of the body. Encased within the hydrophilic exterior is a hydrophobic cavity. This cavity is designed to encapsulate the drug molecule.

Recent research has increasingly highlighted the advantages of using cyclodextrin for the encapsulation of volatiles and EOs. This “magic molecule” enhances the physicochemical steadiness of EOs as volatile compounds, improves the solubility of lipophilic molecules, allows for the controlled release of bioactive materials, and enables the transformation of liquid and oily substances into powder form. In this context, Gharib et al. developed both conventional liposomes (CLs) and drug-in-cyclodextrin-in-liposomes (DCLs) containing estragole through the ethanol injection method, utilizing hydrogenated phospholipids and cholesterol (Figure 6). The study demonstrated that the inclusion of cyclodextrin improved the encapsulation efficiency and oil loading capacity of the liposomes without altering their size. Furthermore, the presence of HP-β-CD (hydroxypropyl-beta-cyclodextrin) or estragole did not impact the size, polydispersity index (PDI), or zeta potential of the estragole DCLs, even after freeze-drying, indicating the stability of these formulations [201].

### 6.2. Physicochemical Properties of Carriers and Their Impact on Skin Penetration

The efficacy of topical delivery systems for plant-derived antimicrobial agents is highly influenced by key physicochemical properties of the carriers, including particle size, zeta potential, and hydrophobicity. These factors determine the stability, skin penetration, and therapeutic performance of the encapsulated compounds.

#### 6.2.1. Particle Size

Smaller NPs (< 100 nm) exhibit enhanced skin penetration due to their ability to traverse the stratum corneum via intercellular or follicular pathways. Larger particles (> 300 nm) are typically retained on the skin surface, limiting deeper delivery. For Example, SLNs and nanoemulsions with droplet sizes below 200 nm have demonstrated improved permeation and sustained release of EOs into the dermis [176].

#### 6.2.2. Zeta Potential

A high absolute zeta potential (> ±30 mV) indicates strong electrostatic repulsion between particles, preventing aggregation and ensuring colloidal stability. Cationic carriers (such as CS-coated NPs) adhere better to the negatively charged skin surface, enhancing retention and permeation [202].

#### 6.2.3. Hydrophobicity

Lipid-based NPs facilitate the delivery of hydrophobic plant compounds (e.g., terpenoids) by integrating into the skin's lipid matrix. Hydrophilic carriers (polymeric hydrogels) are suited for water-soluble agents (e.g., phenolic acids) and provide controlled release [203]. Optimal performance requires balancing these properties. For instance, a nanoemulsion with small droplet size (∼50 nm) and moderate hydrophobicity can enhance EO delivery, while a positive zeta potential (+20 mV) improves skin adhesion [187].

### 6.3. Role of Nanocarriers in Enhancing Antimicrobial Efficacy

The combination of plant-derived antimicrobials with advanced delivery systems offers a synergistic approach to enhancing their therapeutic efficacy. These delivery systems not only address the inherent limitations of plant compounds, such as poor stability, volatility, and low bioavailability, but also amplify their antimicrobial activity through controlled release, targeted delivery, and enhanced penetration. One of the most significant advantages of encapsulating plant-derived antimicrobials in lipid-based NPs is the stabilization of volatile compounds, such as EO. The lipid matrix of these NPs provides a protective environment for the active compounds, ensuring sustained release and prolonged therapeutic effects at the site of infection. Similarly, liposomes have been employed to encapsulate hydrophilic and hydrophobic plant compounds, such as flavonoids and alkaloids, improving their solubility and skin penetration. The phospholipid bilayer of liposomes mimics the structure of cell membranes, facilitating the fusion with microbial cell membranes and enhancing the delivery of antimicrobial agents. For instance, liposomal formulations of berberine, an isoquinoline alkaloid, have demonstrated improved antimicrobial activity against MRSA by increasing intracellular drug concentrations and disrupting bacterial biofilms [204]. The incorporation of *Zataria multiflora* (*Z. multiflora*) EO into CS-based hydrogels has been shown to enhance its antifungal activity against *Candida albicans* [203]. The synergistic effect of the hydrogel matrix and the essential oil results in improved therapeutic outcomes compared to the use of either component alone. Moreover, MN technology has been utilized to deliver plant-derived compounds directly to the deeper layers of the skin, bypassing the stratum corneum [205].

## 7. Advanced Topical Delivery Systems: Nonlipid-Based Topical Delivery Systems

### 7.1. Polymeric-Based Systems

The development of formulations that allow for controlled release of antimicrobial agents can provide sustained therapeutic levels at the site of action [206]. Techniques include the use of polymers that swell or degrade in response to skin conditions or the incorporation of agents into biodegradable matrices [207].

**Figure 7:**
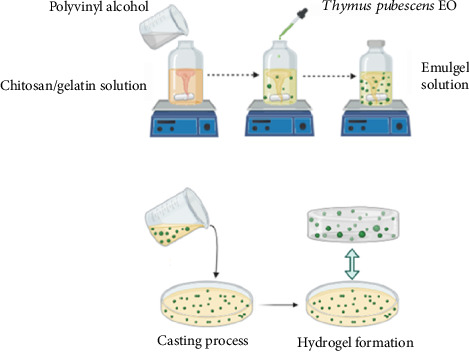
This figure outlines the steps involved in preparing a hydrogel film from an emulgel that contains *Thymus pubescens* EO. The emulgel is an emulsion within a hydrogel matrix, and the film is prepared using the film casting method. Preparation of emulgel: The first step involves formulating the emulgel, which combines the EO with a hydrogel matrix to form a stable emulsion. This creates a system where the EO is uniformly distributed within the gel structure. Film casting: The emulgel mixture is then poured onto a casting surface (such as a flat plate), where it is evenly spread to form a thin layer. Drying and hardening: The film is left to dry at room temperature or under controlled conditions, allowing the water content to evaporate and the hydrogel matrix to solidify, forming a flexible hydrogel film.

In one study, Karami et al. investigated the potential of a CS/gelatin/polyvinyl alcohol (PVA) hydrogel film containing *Thymus pubescens* EO to treat microbial infected wounds (Figure 7). They examined the physicochemical properties, release profile, and biocompatibility of the prepared films. Moreover, the assessment of the antimicrobial and antibiofilm activity of the produced formulations using the broth microdilution and XTT test, respectively, was performed. The formulation developed exhibits remarkable antimicrobial activity against *Escherichia coli*, *Staphylococcus aureus*, *Pseudomonas aeruginosa*, and various *Candida* species.

The synergistic interaction between CS and *Thymus pubescens* EO within the emulgel significantly enhances its efficacy, particularly against Gram-negative bacteria. It is also confirmed that the obtained film can decrease (∼80%) *Candida albicans* biofilm formation and its biocompatibility is verified with hemolysis and MTT (∼100%) analyses. Moreover, the EO-infused film demonstrates an advantageous release profile for *Thymus pubescens* EO, crucial for the effective treatment of infected wounds [202].

Also, Hashempur et al. synthesized and evaluated a creatine-gelatin cryogel enriched with *Z. multiflora* EO and titanium dioxide NPs for potential wound dressing applications. Their findings suggest that this creatine-gelatin cryogel blend possesses several desirable properties: structural integrity, cell-friendly nature, moisture-retaining capabilities, and biocompatible characteristics. These collective properties make the formulated cryogel an exceptionally suitable material for advanced wound dressings [7].

Matshetshe et al. carried out a study where they created a binary system combining β-cyclodextrin-modified CS NPs using the ionic gelation method. This approach aimed to enhance the stability of *Cinnamomum zeylanicum* L. (Darchini) EO and reduce its volatility by encapsulating it. The procedure yielded positively charged, spherical particles with a high EO encapsulation efficiency (greater than 50%). These particles were capable of controlling and sustaining the release of the EO for up to 120 h, showing a significant improvement over the single EO-loaded CS-NP system. The developed system not only augmented the therapeutic properties of the EO but also extended its shelf life, potentially enhancing patient compliance [208].

In their effort to combat bacterial biofilms, which are notoriously resistant to traditional antimicrobial agents, Wang et al. developed CS micelles infused with thymol oil (T-TCP) designed for a controlled, light-triggered release of the antimicrobial substance into the biofilm [209]. These micelles were synthesized through the self-assembly of an amphiphilic copolymer made from toluidine blue O-grafted CS and poly (propylene sulfide). Toluidine blue O is recognized for its ability to generate ROS upon light activation, making it an effective antimicrobial photosensitizer. The effectiveness of T-TCP in adhering to bacterial biofilms was attributed to the electrostatic interactions between the cationic groups of CS and the anionic surface of the biofilm. This interaction led to changes in the biofilm's membrane permeability and barrier functions. The biofilm-binding ability of T-TCP micelles was verified using fluorescence detection. Structurally, the EO-loaded micelles were spherical and exhibited a positive charge of 45.3 mV due to the amine groups on CS, achieving a thymol content of 9.7% (w/w) and an encapsulation efficiency of 30.8%. Exposure of the fluorescein-loaded T-TCP to light for 5 min resulted in a 70% release of the EO after 30 h, a rate three times higher than that without light exposure (20%). Upon light irradiation, T‐TCP micelles produced substantial ROS, resulting in two distinct effects: (1) micelle disassembly (confirmed by scanning electron microscopy) triggered thymol release, and (2) ROS-mediated bactericidal activity disrupted the biofilm. Antibiofilm assays demonstrated that EO-loaded T-TCP micelles successfully eradicated *Listeria monocytogenes* and *Staphylococcus aureus* after 24 h of treatment, outperforming the modest effects of T-TCP alone. This synergistic antimicrobial activity suggests the potential for the developed system to serve as an effective topical disinfectant in the future [209].

### 7.2. Metal NPs

The use of targeted delivery systems, such as NPs engineered to seek out specific microbial pathogens or inflamed tissue, could significantly improve the efficacy of plant-derived antimicrobials [210]. By directing these compounds precisely where they are needed, it may be possible to reduce dosages, minimize side effects, and overcome resistance mechanisms [211].

Nanotechnology offers a promising approach to enhancing the therapeutic potential of plant-derived antimicrobials by enabling targeted delivery systems. Localized and specific administration of antimicrobial agents using this technique could potentially slow down the emergence of resistant strains by lowering selective pressures on microorganisms. Inorganic NPs consist of metals and metal oxides, including silver (Ag), copper oxide (CuO), gold (Au), iron (Fe), platinum (Pt), titanium oxide (TiO_2_), zinc oxide (ZnO), and iron oxide (Fe_3_O_4_) [212, 213]. Inorganic NPs have been extensively researched for several therapeutic purposes, including anticancer and antibacterial treatments. Inorganic pharmaceuticals, like organic medications, can also be upgraded using nanotechnology drug delivery systems (NDDSs) to improve their pharmacokinetic features, including targeted delivery, drug loading efficiency, and evasion of the immune system [214].

The antibacterial properties of metal NPs are influenced by their shape and size. They work by binding to and altering the function of polymers, as well as generating free radicals via ROS. Metallic NPs are viewed as promising options for antibacterial treatments as they may target many cell components, such as cell walls, DNA, membranes, and proteins [215]. Green-synthesized metallic and metal oxide NPs are seen as a promising method to combat germs as a substitute for antibiotics [216]. Nanoconjugates are of interest due to their enhanced biological effectiveness compared to antibiotics in their free form. Ghaffar et al. generated CuO NPs, ZnO NPs, Ag NPs, and FeO NPs using *Ricinus communis* leaf extract. This study demonstrates that *Staphylococcus aureus*, resistant to streptomycin, becomes vulnerable to the same antibiotic when used in conjunction with NP. The NPs combined with antibiotics have synergistic antibacterial effects, offering a new method to reduce drug resistance and potential uses in medicine. Biogenic metallic and metal oxide NPs can efficiently attack multidrug-resistant strains (MDRSs) [217].

### 7.3. Casting Films and Nanofiber (NF) Mats

**Figure 8:**
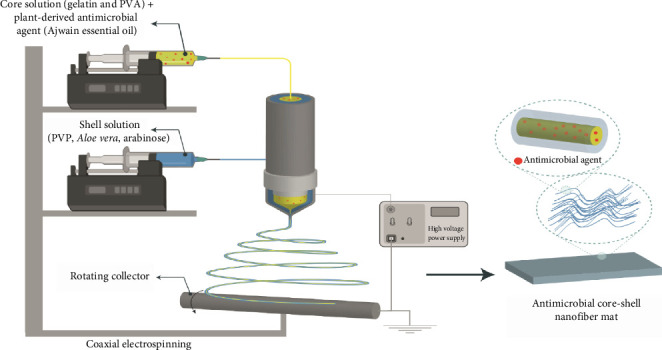
This figure illustrates an electrospinning setup designed to fabricate antimicrobial core-shell nanofiber mats, a technique widely used in drug delivery and other applications. The process begins with a syringe pump, which precisely controls the flow rate of the polymeric solution containing core and shell materials. The solution is loaded into a syringe with a needle, where the needle size and applied voltage determine the resulting nanofiber diameter. A high-voltage power supply charges the polymer solution, forming a charged jet as it exits the needle. Finally, a grounded collector plate attracts and deposits the nanofibers, forming a nonwoven mat structure. This method enables the production of uniform, functional nanofibers with potential antimicrobial and drug-delivery applications.

Recently, nanofibrous scaffolds have emerged as a novel type of wound dressing designed to shield wounds from microbial invasion, maintain a moist environment conducive to healing, allow for oxygen permeation, and facilitate the controlled release of bioactive agents [218, 219]. In this regard, Barzegar et al. developed core-shell nanofibrous scaffolds composed of CS/PVA (as a core) and polyvinylpyrrolidone (PVP)/maltodextrin (MD) (as a shell) for wound healing. The researchers encapsulated EO from *Satureja mutica* or *Oliveria decumbens* within the core of electrospun scaffolds, creating a core-shell structure. The antimicrobial results demonstrated that scaffolds containing *Oliveria decumbens* or *Satureja mutica* EO effectively inhibited the growth of both Gram-positive and Gram-negative bacteria as well as *Candida* species. This broad-spectrum antimicrobial activity could potentially protect the wound site from infection [220]. Zare et al. engineered a NF mat using gelatin and PVA to form the core while incorporating *Aloe vera*, arabinose, and PVP into the shell, aiming to speed up the healing of wounds infected by bacteria (Figure 8) [221]. To leverage the natural antimicrobial properties of *Ajwain* EO against a spectrum of microorganisms, they embedded it within the core of the NF mats through coaxial electrospinning. This integration endowed the core-shell NF mats with robust antimicrobial capabilities against both Gram-positive and Gram-negative bacteria, as well as fungi. Evaluations of in vivo antibacterial activity, wound closure rates, and histomorphological assessments demonstrated the superior effectiveness of the core-shell/EO mat in healing full-thickness rat wounds infected with *Staphylococcus aureus* compared to traditional gauze treatment. These findings highlight the core-shell/EO mat's promise as an innovative wound dressing for treating bacterial infections in full-thickness skin injuries [221]. In distinct research, Liu et al. introduced the concept of zein NF mats encapsulating *Thyme* EO via the in situ electrospinning technique, effectively allowing for direct deposition onto wound sites [222]. This method significantly inhibits the growth of *Escherichia coli* and *Staphylococcus aureus*, thereby preventing infection and facilitating wound healing. Notably, these NFs exhibited super hydrophilic properties, enabling them to efficiently absorb wound exudates.

The findings validated the anticipated positive outcomes of the system, including its bactericidal effectiveness, super hydrophilicity, wound healing promotion, and noncytotoxicity, with more than 90% fibroblast viability [223]. In a related exploration, Mouro et al. applied emulsion electrospinning (utilizing a needle-free nanospider technique) to create NF mats infused with eugenol, incorporating a blend of polycaprolactone (PCL) and PVA with CS. The in vitro eugenol release profile and antibacterial activity against *Pseudomonas aeruginosa* and *Staphylococcus aureus* were also evaluated. The in vitro assessments of these mats revealed controlled EO release over a duration of 120 h, significant bactericidal and bacteriostatic effects against *Staphylococcus aureus* and *Pseudomonas aeruginosa*, a high rate of bacterial reduction (greater than 80%), and the notable viability of normal human dermal fibroblasts across 7 days [224]. In another study, Lamarra et al. explored the incorporation of cabreuva EO into NPs crosslinked with CS, which were then embedded into PVA-based NFs through the electrospinning technique [225]. The antibacterial capacity of the NFs was also investigated, as well as their ability to modulate the controlled release of EO in various media. This novel system demonstrated a controlled release pattern for the encapsulated EO, along with significant antimicrobial activity against several pathogens, including *Candida albicans*, *Staphylococcus aureus*, *Staphylococcus epidermidis*, and *Escherichia coli*. Consequently, the cabreuva EO-infused NF presents itself as a versatile platform, offering potential applications as either a controlled release matrix or a scaffold for tissue engineering purposes [225].

### 7.4. Hydrogels

Hydrogels are an appropriate choice for a delivery platform when it comes to enhancing the stability of EOs, given their high volatility. Adhesive hydrogel patches act as viscoelastic structures with a high liquid-to-polymer mass ratio, closely mimicking the composition of native tissue constructs [7, 202, 203]. The ability to retain a high amount of water is particularly crucial in applications aimed at healing wounds, where it is necessary to absorb substantial volumes of wound exudate [202]. Their porous structure allows for the controlled release of antimicrobial agents, making them ideal for treating skin infections and promoting tissue regeneration. The polymers that make hydrogels can be of synthetic or natural origin, featuring biocompatible and biodegradable qualities with physicochemical properties that can be readily adjusted [203].

In one study, Torabiardekani et al. encapsulated *Z. multiflora* EO as an herbal therapeutic agent in a PVA/CS/gelatin hydrogel as a positive thermoresponsive and antifungal platform [203]. This innovative and noninvasive hydrogel shows promise in delivering *Z. multiflora* EO at body temperature, exhibiting both antifungal properties and biocompatibility. Furthermore, the EO, when administered through this hydrogel, proves to be more effective than in its pure form. This enhances the potential for a new drug delivery method by lowering the required concentration of therapeutic agents in the target tissues. Therefore, the developed hydrogel, characterized by its biocompatibility, antifungal efficacy, and positive thermoresponsive behavior, holds potential as a topical antifungal treatment for cutaneous candidiasis [203]. In another study, Alsakhawy et al. encapsulated thyme EO in sodium caseinate (Na CAS) nano micelles and formulated a gelatin nanocomposite hydrogel [226]. The release dynamics of thyme EO-loaded nanocomposite hydrogel demonstrated a more sustained release pattern when contrasted with thyme EO-loaded micelles and the free oil itself. Notably, the thyme EO encapsulated within nano micelles showed markedly enhanced antibacterial activity as evidenced by increased leakage of alkaline phosphatase and disruption of cell membranes compared to the free thyme EO. Moreover, the thyme EO-loaded nanocomposite hydrogel was significantly effective in promoting wound contraction, reducing levels of interleukin-6, and elevating the production of transforming growth factor-β1 and vascular endothelial growth factor, as compared to either the control group or those treated with a blank hydrogel. This underscores the hydrogel's potential for advancing wound healing and reducing inflammation [226]. Carboxymethyl CS-based hydrogels were created and infused with selected EOs, including eucalyptus, ginger, and cumin, to produce antibacterial materials. Among these hydrogels, the one loaded with *eucalyptus* EO showed superior antibacterial performance, achieving inhibition rates of 46% against *Staphylococcus aureus* and 63% against *Escherichia coli*. This eucalyptus-infused hydrogel also demonstrated high cell viability (over 92%) and significantly enhanced wound healing in mouse burn models by facilitating the regeneration of both dermis and epidermis layers [227, 228].

Zhong et al. developed injectable self-healing hydrogels based on CS, which possess intrinsic antibacterial properties, through the formation of dynamic boronate ester cross-linkages. Additionally, they achieved in situ encapsulation of epigallocatechin-3-gallate, a compound derived from green tea. These innovative hydrogels exhibited dual bioactivity, displaying both antibacterial effects against *Escherichia coli* and *Staphylococcus aureus* and antioxidant properties, along with notable biocompatibility. Further, in vivo wound healing experiments using a skin model demonstrated the hydrogels' effectiveness, highlighting their promise as wound dressing materials [229]. Burn injuries present a unique challenge that requires the creation of specialized therapeutic materials with antibacterial properties to combat the infectious microbes commonly found in burn wounds [7]. A thymol-enriched bacterial cellulose hydrogel has proven to be an effective solution for burn wound dressings. It demonstrates significant antibacterial efficacy against pathogens such as *Escherichia coli, Staphylococcus aureus*, *Pseudomonas aeruginosa*, and *Klebsiella pneumoniae.*

Additionally, this hydrogel has shown promising in vivo wound healing capabilities in rats with third-degree burns, indicating its potential as a beneficial treatment option for burn injuries [230]. Tran and colleagues devised an innovative approach for treating dermal wounds with the development of a rutin (RU)-conjugated CS hydrogel, envisioned as an injectable wound dressing. This RU-conjugated CS-polyethylene glycol-tyramine (RU-CS-PEG-TY) polymer, in the presence of horseradish peroxidase (HRP) and hydrogen peroxide (H_2_O_2_), forms an in situ gel. This novel hydrogel has been shown to significantly enhance wound healing in male Sprague-Dawley rats, showcasing its potential as an effective solution for wound care (*p* < 0.05) [231].

### 7.5. Plant Mucilages

Plant mucilage is a hydrocolloid that consists mostly of amino acids, carbohydrates, monosaccharides, uranic acid units, and glycoproteins. The amounts and compositions of these components vary depending on the plant's origin. Mucilage is an essential component of plant cells and is found in several regions of the plant, including the seeds [232, 233]. Humans have traditionally utilized mucilage as a texturing and gelling product in the industry. Over time, it has shown its value and cost-effectiveness as a natural component supply in the biomedical area. It is now utilized as a biocompatible, biodegradable, and nontoxic substance that controls the rate and forms a matrix in TDDSs and wound dressing applications [234]. Initially, in the development of drug delivery systems, plant mucilages were utilized as mucoadhesive polymers because of their chemical properties, which include a high number of hydroxyl and carboxyl groups. Studies on *Mimosa pudica* mucilage from the Mimosaceae family demonstrated beneficial impacts on drug release percentage and bioadhesion time [235]. Recent reports have shown similar positive findings regarding the mucilage of *Datura stramonium* leaves. Ahad et al. discovered in a factorial investigation that the concentration of mucilage affects the pace and amount of aceclofenac release, as well as the adhesion time [236]. Plant mucilage's characteristics, particularly its charge density, are enhanced using electrospinning, a recent process used to produce NFs. Nanofibrillated mats as well as patches are seen as excellent materials for wound healing due to their ability to provide adequate oxygen supply and easy integration of the API [237]. *Plantago ovata* mucilage was utilized as a precursor in the production of core-shell NFs. Biodegradable core-shell NFs that respond to stimuli were created by employing mucilage as the outer shell solution and paclitaxel (PTX)-loaded PCPP-CA micelles as the inner core solution [238]. The intricate nanomaterial, structured as a patch, was created to enable the regulated transdermal distribution of PTX for breast cancer treatment. Lab experiments showed that there was a pH-responsive and prolonged drug release lasting 180 h, resulting in enhanced anticancer effectiveness compared to free PTX. A novel mixture of PVA, *Hibiscus rosa-sinensis* leaves' mucilage (HLM), and pectin was electrospun and crosslinked similarly using glutaraldehyde vapor [237]. The green mat produced can serve as a scaffold of nanofibrillated polymeric material for the healing of wounds. This is an illustration of how synthetic and natural polymers may be mixed to create a biodegradable and hemocompatible product. Between 2010 and 2020, the utilization of plants' mucilage was infrequent and had limited application. However, there has been a recent increase in research interest due to a trend toward natural products. Modern methods of extraction, isolation, characterization, and analysis have enabled the discovery of secondary metabolites in mucilages, particularly those derived from a plant's leafy sections. In certain instances, plant mucilage serves as a biopolymer with pharmacological properties such as antibacterial and antioxidant effects, while in other circumstances, it acts as the API in a more intricate structure [239].

### 7.6. MN for Enhanced Penetration

MN technology, which involves the use of tiny needles to create MN in the skin, holds great promise for the transdermal delivery of plant-derived antimicrobials [205]. This method can be particularly beneficial for the systemic effects of antimicrobial agents by temporarily disrupting the stratum corneum, where constant plasma levels are required. MN has been widely used for enhancing the transdermal delivery of numerous kinds of drugs [240, 241]. MN can pierce through the stratum corneum of the skin to access the dermis, all while avoiding damage to nerve endings or blood vessels within the dermal layer. The latest advancement in MN technology involves the development of hydrogel-forming MN [242]. This technology diverges from the conventional method of blending drugs with polymers by employing a reservoir filled with the drug, which is subsequently combined with blank MN during application. The distinction lies in the ability of the MN to absorb significant amounts of interstitial fluid post-insertion into the skin, causing it to swell within the skin tissue [242]. This approach can significantly enhance the penetration of these compounds, enabling direct access to deeper skin layers and potentially even systemic circulation, without causing significant pain or discomfort [241]. Future research could explore the integration of plant-derived compounds into dissolvable or biodegradable MN patches for sustained release and enhanced therapeutic effects.

### 7.7. Wearable Drug Delivery Systems

The development of wearable drug delivery technologies, such as smart patches that can respond to skin temperature, moisture, or other physiological cues, represents an exciting frontier for dermal delivery [243]. These systems could offer on-demand release of plant-derived antimicrobials, providing personalized treatment regimens based on the patient's specific condition or needs. Investigating the compatibility of plant-derived compounds with such smart materials and systems could open up new avenues for the treatment of chronic skin conditions. Wearable drug delivery systems have a chance to change how we take medicine, especially in the context of dermal delivery. The idea of smart patches that can react to different physiological markers like skin temperature and moisture is one step closer to achieving individualized treatment [244]. However, by including plant-based antimicrobials, it may be possible to provide specific therapies for chronic skin conditions through such technologies. Consequently, there will be higher success rates in medicine as well as fewer side effects due to fewer general prescriptions and an increase in patients following their prescriptions [245]. A comparative analysis of lipid-based and nonlipid-based delivery systems is presented in Table 1.

## 8. Advantages and Limitations of Plant-Based Carriers

### 8.1. Skin Barrier Properties

The skin serves as a critical barrier, protecting the body from external threats while maintaining homeostasis [246]. This role presents challenges for the topical delivery of therapeutic agents, including plant-derived antimicrobial compounds, as the stratum corneum, the skin's outermost layer, is notably impermeable due to its composition of densely packed dead cells and lipids [247]. Overcoming this barrier without causing damage or irritation necessitates innovative approaches such as the use of penetration enhancers, which temporarily alter the stratum corneum's lipid structure, and physical methods like MN, which improve the delivery of active compounds into deeper skin layers. Additionally, nanotechnology, including NPs and liposomes, helps bypass the skin barrier by controlling the transport of encapsulated drugs [248].

### 8.2. Safety and Toxicity

While plant-derived antimicrobial agents are often considered safer alternatives to synthetic drugs, they also pose risks, including safety and toxicity concerns [249]. Natural compounds can provoke allergic reactions or contact dermatitis in sensitive individuals, and there is a potential for systemic toxicity [250], especially with compounds more readily absorbed through the skin [251]. This highlights the importance of comprehensive toxicity evaluations to ensure the safety of these antimicrobials and their delivery systems, including assessments of cytotoxicity, genotoxicity, and long-term health effects [252]. Moreover, the development and commercialization of plant-derived therapeutics encounter significant regulatory and standardization challenges [253]. The variability in the concentration and composition of active compounds in plant extracts can affect efficacy and safety, necessitating standardized extraction processes and quality control measures [11].

### 8.3. Regulatory and Standardization Issues

**Figure 9:**
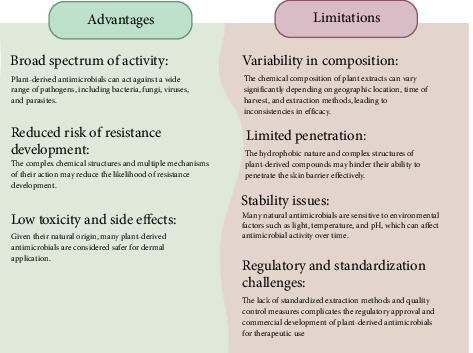
Compares the key advantages and limitations of plant-based carriers for delivering antimicrobial agents. Among the advantages, plant‐derived antimicrobials exhibit broad-spectrum activity against bacteria, fungi, viruses, and parasites. Their complex chemical structures and multiple mechanisms of action may reduce the likelihood of microbial resistance compared to conventional antibiotics. Additionally, their natural origin often results in lower toxicity and fewer side effects than synthetic alternatives. However, several limitations exist, including variability in composition due to factors like geographic origin, harvest time, and extraction methods, which can impact consistency and efficacy. Some compounds also face limited penetration due to their hydrophobic nature, reducing their ability to cross biological barriers. Stability issues arise from sensitivity to light, temperature, and pH, complicating storage and long-term effectiveness. Finally, regulatory and standardization challenges, such as inconsistent extraction methods and quality control, hinder the approval and therapeutic use of plant-based antimicrobials.

Navigating the regulatory landscape is complex, with stringent requirements for demonstrating safety, efficacy, and quality, and variability in regulations across different jurisdictions complicating the approval process for new therapeutics. Ensuring batch consistency is another challenge due to the natural variability in raw plant materials, requiring rigorous cultivation, harvesting, and extraction protocols [254]. Addressing these challenges is crucial for the successful development and application of plant-derived antimicrobial agents in dermal drug delivery. Innovations in formulation technology and rigorous scientific and clinical evaluation are necessary to overcome skin barriers and ensure the safety and efficacy of these natural compounds. Furthermore, standardization and regulatory compliance are essential for gaining acceptance and trust in these therapeutics, paving the way for their integration into mainstream medical practice. Some of the advantages and limitations of plant-based carriers are mentioned in Figure 9.

#### 8.3.1. Stabilization Methods to Prevent Degradation of Agents Derived From Plants

Antimicrobial agents derived from plants are often susceptible to degradation by light, temperature, or pH changes. To address these challenges, various stabilization methods have been developed, including the use of antioxidants, chelating agents, and encapsulation techniques. Each method offers distinct advantages and limitations, making them suitable for different applications.

##### 8.3.1.1. Antioxidants

Antioxidants, such as ascorbic acid [255], tocopherols [256], and polyphenols [257], are commonly used to prevent oxidative degradation of plant-derived antimicrobial agents. They work by scavenging free radicals and inhibiting oxidation reactions. While antioxidants are cost-effective and easy to incorporate, their efficacy is often limited to specific environmental conditions and may require high concentrations to achieve significant stabilization. Specific examples include:

a. Ascorbic acid (vitamin C): Ascorbic acid has been shown to significantly enhance the stability of EOs in topical formulations. Youce Ettoumi et al. showed that the antioxidant activity of the orange EO emulsion combined with ascorbic acid was significantly higher than that of the emulsion without ascorbic acid and greater than that of the individual components [258].
b. Tocopherols (vitamin E): Tocopherols are effective in preventing lipid peroxidation in EO-based formulations. Zhang et al. studied the effects of tocopherol and chlorogenic acid on the oxidation stability of *algal* EO emulsion. Their findings indicated that the combination of tocopherol and chlorogenic acid effectively inhibited the oxidation of the *algal* EO emulsion [259].
c. Polyphenols: Polyphenols are natural antioxidants that also exhibit antimicrobial properties. Nawaz et al. reported that curcumin hydrogels formulated with natural essential oils may provide an effective solution for skin infections and wound healing. Curcumin, a polyphenol derived from turmeric, is the key component in these hydrogels [227, 260].

##### 8.3.1.2. Chelating Agents

Chelating agents, such as ethylenediaminetetraacetic acid (EDTA) [261] and citric acid [262], stabilize plant-derived compounds by binding metal ions that catalyze degradation reactions. These agents are particularly effective in preventing metal ion–induced oxidation. However, their use is restricted in some applications due to regulatory concerns and potential toxicity at high concentrations. Specific examples include:

a. EDTA: EDTA is a widely used chelating agent in topical formulations [263]. For example, EDTA has been shown to enhance the oxidative stability of fish oil emulsions and spray-dried microcapsules. It generally reduces oxidation by minimizing the formation of volatile compounds, which are indicators of lipid oxidation [264]. Another study reported that EDTA's effectiveness in reducing lipid oxidation in O/W emulsions is attributed to its ability to chelate metal ions, which are often involved in initiating oxidation reactions. This makes EDTA a valuable additive in formulations where oxidative stability is crucial [265].
b. Citric acid: Citric acid is a natural chelating agent that is often used in combination with antioxidants. It is known for its ability to chelate metal ions, which can catalyze oxidation reactions [266]. This property makes it useful in formulations where metal ion control is necessary to prevent degradation. Citric acid is often combined with antioxidants like ascorbic acid to enhance their effectiveness. For example, the combination of citric acid and ascorbic acid has been shown to be highly effective in controlling oxidation in food products, such as reducing corruption in frozen fish fillets [267].
c. Phytic acid: Phytic acid is a natural chelating agent derived from plant seeds [260]. Lombardo et al. demonstrated that phytic acid provides protection against oxidative stress caused by genetic iron overload, whether occurring alone or in conjunction with a high-fat diet [263]. In another report, the effect of antioxidant phytic acid on the stability of Pickering emulsions was studied by Polavarapu et al. [264].

##### 8.3.1.3. Encapsulation Methods

Encapsulation within emulsion-based systems, such as microemulsions and nanoemulsions, is a more advanced and versatile approach [268, 269]. These systems protect sensitive compounds from environmental factors like oxidation, light, and high temperatures. Microemulsions offer high stability and ease of preparation but may require high surfactant concentrations. Nanoemulsions, on the other hand, provide superior physical stability, controlled release, and enhanced bioactivity due to their nanoscale droplet size. However, they often involve more complex formulation processes and higher production costs. Among these methods, encapsulation within nanoemulsions is particularly promising for enhancing the stability and efficacy of EOs. This approach not only reduces the volatility of EOs but also extends their release duration and improves their bioactivity. A comparative summary of these stabilization methods is provided in Table 2, highlighting their key characteristics and applications.

## 9. Experimental Applications of Plant-Based Topical Formulations

Recent clinical studies have demonstrated the therapeutic potential of plant-based topical formulations in wound healing, showcasing their efficacy in comparison to conventional treatments.

One notable clinical trial investigated the wound-healing effects of a CS-based hydrogel loaded with *Z. multiflora* EO nanoemulsions for the treatment of chronic wounds [272]. The study utilized a PCL-CS scaffold coated with a gel containing *Z. multiflora* EO nanoemulsions and evaluated its impact on wound closure rates. The results revealed a significant improvement in wound healing compared to standard care, attributed to the synergistic effects of CS's biocompatibility and the antimicrobial and anti-inflammatory properties of *Z. multiflora* EO. This formulation not only enhanced tissue regeneration but also reduced the risk of infection, making it a promising alternative for chronic wound management [274].

Another randomized controlled trial explored the combined effects of Ag NPs and *Aloe vera* extract in burn wound healing [273]. The study, conducted on a rat model, demonstrated that the application of Ag NPs combined with *Aloe vera* gel significantly accelerated tissue regeneration compared to conventional treatments. *Aloe vera* contributed to its well-known moisturizing, anti-inflammatory, and collagen-stimulating properties, while Ag NPs provided potent antimicrobial activity, preventing infection and promoting faster wound closure. The findings suggest that this combination therapy could be an effective strategy for enhancing burn wound recovery [275].

Table 3 summarizes FDA-approved plant-based topical products, including Veregen® (green tea extract) for genital warts and Abreva® (docosanol) for cold sores, highlighting their formulations, therapeutic uses, and approval status. These products demonstrate the clinical viability of botanical components, with Veregen® and Abreva® serving as key examples of plant-derived therapeutics in dermatology. The table underscores the FDA's recognition of plant-based treatments, offering targeted efficacy for specific conditions while reflecting advancements in natural product drug development.

## 10. Future Directions and Research Gaps

Despite the vast biodiversity of plants, only a fraction has been explored for their antimicrobial potential, highlighting the need for systematic screening of traditional medicinal plants to discover novel compounds with unique mechanisms of action [278] requiring collaborative efforts across multiple disciplines. While delivery system advancements continue, optimization remains crucial for improving stability, solubility, and skin penetration of plant compounds, along with developing multifunctional systems for complex skin infections [279] that integrate traditional knowledge with modern technologies [280]. Emerging biotechnological tools like CRISPR-Cas9 gene editing show promise for enhancing plant-derived antimicrobials through precise modification of biosynthetic pathways to increase production of key compounds (e.g., thymol in *Thymus vulgaris*), create novel derivatives with enhanced activity, and improve stability for topical formulations though challenges remain in regulatory pathways, standardization, and safety evaluation. These CRISPR-optimized metabolites could synergize with advanced delivery systems by providing higher-purity actives, enabling tailored physicochemical properties, and supporting scalable production. Realizing the full therapeutic potential will require cross-disciplinary collaboration, continued innovation in both bioactive discovery and delivery technologies, and a balanced approach combining traditional knowledge with modern scientific methods [281, 282].

### 10.1. Innovative Research Directions for Plant-Based Topical Delivery

While significant progress has been made in the development of plant-derived antimicrobials and advanced delivery systems, several innovative research directions hold promise for further advancing the field. These forward-looking approaches not only address current limitations but also open new avenues for personalized and precision medicine in topical drug delivery.

#### 10.1.1. Artificial Intelligence (AI)–Driven Design for Optimizing Plant-Based Formulations

The integration of AI and machine learning into the design and optimization of plant-based formulations represents a transformative approach. AI algorithms can analyze large datasets on plant metabolites, their physicochemical properties, and interactions with delivery systems to predict optimal formulations. AI-driven models can identify the most effective combinations of plant compounds and nanocarriers for specific skin infections, reducing the time and cost associated with experimental screening. Additionally, AI can be used to optimize parameters such as particle size, encapsulation efficiency, and release kinetics, ensuring maximum therapeutic efficacy [283].

#### 10.1.2. 3D-Printed MNs for Personalized Delivery

The advent of 3D printing technology has revolutionized the fabrication of MNs, enabling the creation of highly customizable and patient-specific delivery systems. 3D-printed MNs can be tailored to deliver precise doses of plant-derived antimicrobials based on individual patient needs, such as skin type, infection severity, and treatment duration. This personalized approach not only enhances therapeutic outcomes but also improves patient compliance. Furthermore, 3D printing allows for the incorporation of multiple active ingredients into a single MN patch, enabling combination therapies that target multiple pathways of microbial resistance [284, 285].

## 11. Conclusion

This review has systematically explored the developing landscape of plant-derived antimicrobial agents and their application in topical drug delivery, underscoring the significant potential these natural compounds hold in addressing skin infections and conditions. The investigation of different classes of plant-derived antimicrobials, including phenolics, alkaloids, terpenoids, and flavonoids, shows a rich variety of composites with potent antimicrobial properties. These agents offer a promising alternative to traditional antibiotics, principally in an era where antimicrobial resistance is a growing alarm. The challenges posed by the skin's protective barrier have highlighted the critical role of advanced delivery systems in enhancing the efficacy of plant-derived antimicrobials. Innovations such as NPs, liposomes, hydrogels, and transdermal patches have emerged as key strategies to overcome these problems, enabling improved penetration, stability, and controlled release of these compounds. The successful outcomes and clinical applications discussed herein provide evidence of the effectiveness of these advanced delivery mechanisms, showcasing significant improvements. Additionally, the interdisciplinary nature of this field, about botany, pharmacology, material science, and nanotechnology, highlights the cooperative efforts in driving innovation and overcoming the complications related to developing plant-derived therapeutics.

## Figures and Tables

**Table 1:** Comparison of lipid-based and nonlipid-based delivery systems.

Parameter	Lipid-based systems	Nonlipid-based systems
Stability	Suitable for hydrophobic compounds (such as EOs and terpenoids).	Better stability for hydrophilic agents (such as plant peptides and phenolic acids).
	Challenges with oxidation of lipids.	Polymeric systems (such as CS and PLGA) offer high stability.

Penetration efficiency	Moderate penetration through the stratum corneum.	Superior penetration with microneedles.
	Limited penetration for large or hydrophilic molecules.	Enhance delivery to deeper skin layers (nanofibers and hydrogels).

Controlled release	Provide sustained release but may have initially burst release.	Offer sustained and controlled release (hydrogels and polymeric nanoparticles).
	Release kinetics depend on lipid composition.	Release profiles can be adjusted by altering polymers (such as crosslinking).

Scalability and cost	Simpler manufacturing processes.	Polymeric systems may require more complex manufacturing.
	Cost-effective (large-scale production).	Higher cost for advanced systems (such as microneedles and nanofibers).

Biocompatibility	Biocompatible but may cause irritation (such as surfactants).	Highly biocompatible (such as CS, PLGA, and PVA).

Applications	Perfect for volatile compounds (such as EOs) and hydrophobic drugs.	Suitable for hydrophilic compounds.

Advantages	High encapsulation efficiency for lipophilic compounds.	Versatile and tunable for a wide range of compounds.
	Enhance stability and bioavailability of plant-derived antimicrobials.	Targeted and controlled delivery.

Limitations	Limited ability to encapsulate hydrophilic compounds.	Complex manufacturing processes for some systems (such as electrospinning).
	Potential for lipid oxidation and degradation.	Higher cost and scalability challenges for advanced systems.

**Table 2:** Comparison of stabilization methods.

Stabilization method	Mechanism	Advantages	Limitations	Applications	Ref.
Antioxidants	Scavenge free radicals	- Cost-effective	- Limited efficiency	- Food	[270]
		- Easy to incorporate	- Require high concentrations	- Cosmetics	
				- Pharmaceuticals	

Chelating agents	Bind metal ions	- Effective against metal ion	- Regulatory concerns	- Food preservation	[271]
		- Induced degradation	- Potential toxicity (high concentrations)	- Pharmaceuticals	

Microemulsions	Encapsulation in surfactant-based systems	- High stability	- High surfactant concentrations	- Topical delivery	[272]
		- Ease of preparation		- Cosmetics	

Nanoemulsions	Encapsulation in nanoscale droplets	- Superior stability	- Complex formulation	- Drug delivery	[273]
		- Controlled release	- Higher production costs	- Topical antimicrobials	
		- Enhanced bioactivity			

**Table 3:** FDA-approved plant-based topical products and their clinical applications.

Product name	Active plant component	Formulation	Therapeutic use	Approval status	Reference
Veregen® (sinecatechins)	Green tea extract (*Camellia sinensis*; catechins)	Ointment (15% w/w)	Treatment of genital warts	FDA-approved (2006)	[276]
Abreva®	Docosanol (derived from sugarcane)	Cream (10%)	Cold sores	FDA-approved (2000)	[277]

## Data Availability

The data that support the findings of this study are available from the corresponding author upon reasonable request.
